# A blood circulation-prolonging peptide anchored biomimetic phage-platelet hybrid nanoparticle system for prolonged blood circulation and optimized anti-bacterial performance

**DOI:** 10.7150/thno.49781

**Published:** 2021-01-01

**Authors:** Peipei Jin, Liansheng Wang, Rui Sha, Liu Liu, Jieying Qian, Nestor Ishimwe, Wenbin Zhang, Jing Qian, Yunjiao Zhang, Longping Wen

**Affiliations:** 1 Department of Laboratory Medicine, The First Affiliated Hospital of USTC, School of Life Sciences, Division of Life Sciences and Medicine, University of Science and Technology of China, Hefei 230036, China; 2 Department of Surgery, Guangzhou First People's Hospital, School of Medicine and Institutes for Life Sciences, South China University of Technology, Guangzhou 510006, China; 3 National Engineering Research Center for Tissue Restoration and Reconstruction, South China University of Technology, Guangzhou 510006, China; 4 Key Laboratory of Biomedical Engineering of Guangdong Province, and Innovation Center for Tissue Restoration and Reconstruction, South China University of Technology, Guangzhou 510006, China; 5 Guangzhou Regenerative Medicine and Health Guangdong Laboratory), Guangzhou 510005, China; 6 China School of Environment and Energy Engineering, Anhui Jianzhu University, Hefei 230061, China

**Keywords:** phage therapy, biomimetic nanoparticle, prolonged blood circulation, hybrid, anti-bacterial

## Abstract

Phage therapy holds great promise for resolving the ever-worsening crisis of antibiotic resistance, but it also faces many challenges. One of the issues hampering phage therapy is the short blood residence time of bacteriophages. We have previously identified, through *in vivo* phage display, a blood circulation-prolonging peptide (BCP1) that was capable of significantly prolonging the blood retention time of a doxorubicin-loaded human ferritin nanocage, leading to enhanced therapeutic efficacy against tumors. Herein, we aimed to extend the application of BCP1 to anti-bacterial phage therapy.

**Methods:** A genetically engineered M13 phage, BCP1-BGL, that displayed the BCP-1 peptide and expressed the restriction endonuclease *Bgl II*, was constructed. Taking advantage of the fact that BCP1 harbors an RGD motif (a three amino-acid sequence Arg-Gly-Asp with the ability to bind to integrins) and exerts its circulation-prolonging activity primarily through interaction with platelets, we further designed and fabricated a biomimetic phage-platelet hybrid nanoparticle (PPHN) *via* the physical binding of the BCP1-BGL phage to the platelet membrane nanoparticles derived *via* a repeated freeze-thaw procedure. A series of experiments *in vitro* and* in vivo* were conducted to reveal the long circulation and anti-bacterial capacities of BCP1-BGL phages and PPHNs.

**Results:** The resulting PPHNs possessed a hydrodynamic size of 368 nm in deionized water, with each spherical membranous nanoparticle harboring approximately 12 rod-shaped phage particles stably bound to its surface. PPHNs, which were superior to the BCP1-BGL phages that displayed significantly prolonged anti-bacterial action *in vivo* against *Escherichia coli* infection, exhibited further extended blood retention time and optimal anti-bacterial performance in both the prophylactic and treatment approaches.

**Conclusion:** Our work demonstrated a novel strategy in engineering biomimetic phage-based nanoparticles with improved blood retention and anti-bacterial performance and may have implications in phage therapy.

## Introduction

Phage therapy, which applies bacteria viruses (bacteriophages) to combat bacterial infections, has been in clinical practice since the early 20^th^ century but was largely abandoned after the discovery of antibiotics 1-3. However, the rising tide of antibiotic resistance, particularly with the emergence of multi-resistant and even pan-resistant bacteria, has sparked a renewed interest in phage therapy in recent years 4, 5. In fact, antibiotic resistance currently constitutes one of the most prevalent global public health concerns 6-8. Antimicrobial resistance is predicted to cause 10 million annual deaths by 2050, costing the society approximately 100-200 trillion US dollars 9,10. Although phage therapy holds great promise for solving the ever-worsening crisis of antibiotic resistance, it also faces many challenges. One of the issues that have hindered the successful clinical implementation of phage therapy is the short blood residence time, as phages will be rapidly cleared from the bloodstream 11,12. Engineering therapeutic bacteriophages with extended blood circulation time are thus a research priority and would enable more effective phage therapy in either curing existing bacterial infections or preventing bacteria re-infection. Several strategies, such as selection through naturally-occurring mutations 13,14 and chemical modification of phage surface by polyethylene glycol 15, have been proposed and, in some cases, led to the development of phage with significantly improved blood retention. However, selection through naturally-occurring mutations was associated with the disadvantage of a long cycle and low efficiency, while chemical modifications cannot be controlled and may interfere with phage activity. Therefore, new innovative strategies are highly desirable for engineering long-circulating bacteriophages with improved anti-bacterial performance.

Biomimetic drug delivery offers new opportunities to mimic biological particulates, including cells, vesicles and viruses for enhancing biocompatibility and promoting therapeutic efficiency 16,17. As a simple and effective biomimetic approach, delivery vehicles coated with cell membranes are extensively researched, and were found to have a variety of merits, such as prolonging the circulation time, alleviating immunogenicity, and accomplishing active targeting. To date, membranes of fibroblast cells, cancer cells, red blood cells (RBCs), white blood cells (WBCs), and platelets (PLT) have all been successfully applied in preparing biomimetic drug delivery systems 18-22. Among these, membranes of PLT, the nonnuclear fragments of mature megakaryocytes cropped by the bloodstream in the sinusoidal vasculature, offer an attractive choice for fabricating biomimetic nanoparticles. Gu et al. developed a PLT membrane-coated core-shell nanovehicle for sequential and site-specific delivery of protein as well as small molecular drugs. The overexpressed P-selectin on the platelet membrane can specifically bind to CD44 receptors upregulated on the surface of cancer cells, leading to active targeting to the tumor site 23. Li et al. coated synthetic silica particles with PLT membranes to deliver TRAIL for capturing and killing circulating tumor cells (CTCs). As expected, the PLT membrane-based nanocarriers showed reduced phagocytic uptake owing to the preservation of the CD47 protein, and efficient elimination capability of CTCs due to the specific binding of PLT membrane to CTCs. Zhang et al. developed the pore-forming toxins (PFTs) nanosponge, which consists of a polymeric nanoparticle core surrounded by red blood cell (RBC) membranes. The RBC membrane shell provides an ideal mimicry to absorb a wide range of PFTs regardless of their molecular structures. In addition, the inner polymeric core stabilizes the RBC membrane shell to enable prolonged systemic circulation essential for absorbing toxins in the bloodstream. This biologically inspired nanosponge was proven to effectively reduce toxicity and improved survival of toxin-challenged mice 24,25.

In our previous study, we have identified, through the innovative use of *in vivo* phage display, a blood circulation-prolonging peptide (BCP1) that was capable of significantly prolonging the blood retention time of doxorubicin-loaded human ferritin nanocages, leading to enhanced therapeutic efficacy against tumors 11. In this report, we have extended the application of BCP1 to anti-bacterial phage therapy. We first engineered an M13 phage, BCP1-BGL, which possessed both long-circulating properties (through displaying the BCP-1 peptide) and bactericidal activity (through expressing the restriction enzyme *Bgl* Ⅱ) and showed that the engineered phage exhibited significantly prolonged *in vivo* anti-bacterial activity against *Escherichia coli* infection. Taking advantage of the fact that the BCP-1 peptide harbored an RGD motif and exerted its circulation-prolonging activity through interaction with peripheral blood cells (PBC), particularly PLT, we further designed a biomimetic phage nanoparticle composed of the PLT membrane and BCP1-BGL, which exhibited further prolonged blood retention and optimized anti-bacterial performance in both the prophylactic and treatment approaches. Our work demonstrated a new strategy in engineering biomimetic phage delivery system with improved anti-bacterial performance and may have implications for phage therapy.

## Results

### Construction of BCP1-BGL, an engineered M13 phage with bactericidal properties and prolonged blood circulation

**Figure 1:**
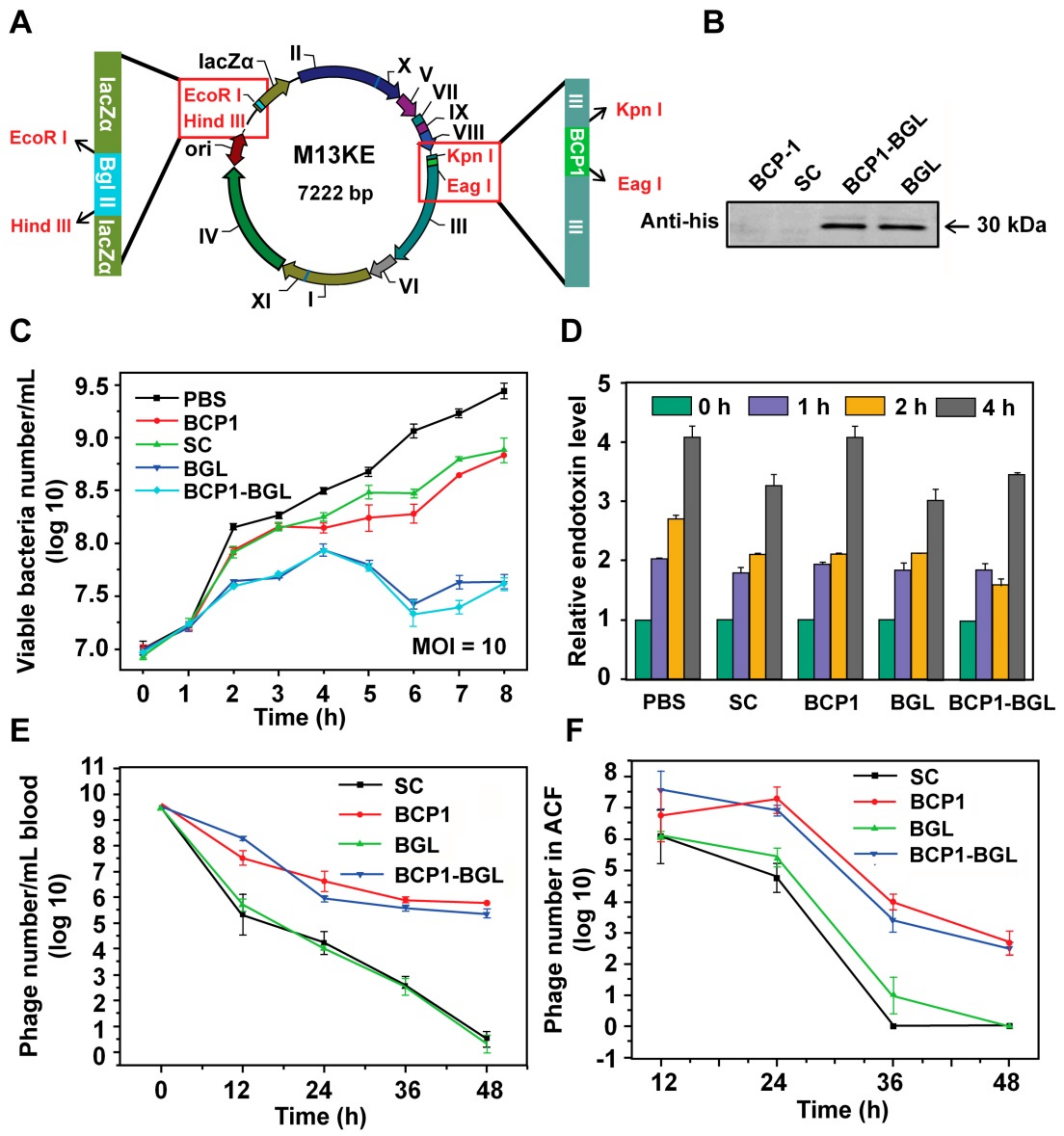
** Engineered long-circulating anti-bacterial phages and their activity *in vitro.***(A) Schematic illustration of long-circulating antimicrobial phage construction. (B) Detection of *Bgl II* protein (30 kDa) with western blotting with anti-his antibody, following phage infection of *E. coli* (OD 600~0.2). (C) Antibacterial activity of various phages *in vitro* at a MOI of 10. The number of viable *E. coli* cells was determined at various time points after phage infection with the colony formation assay. Mean ± S.E.M., n = 3. (D) Endotoxin release. Endotoxin level in the culture supernatant was determined by ELISA at various time points following phage infection of *E. coli*. Mean ± S.E.M., n = 3. (E) Comparison of blood circulation time for BCP1, SC, BGL and BCP1-BGL. The same number (1 × 10^11^ pfu) of individual phages was separately injected into rats with phage titers in the blood determined at various time points. Mean ± S.E.M., n = 3. (F) Enrichment of long-circulating phage in ACF. Mean ± S.E.M., n = 3.

To engineer the M13 phage with antibacterial activity, we inserted a his-tagged *Bgl* Ⅱ gene, which encodes for the *Bgl* Ⅱ restriction endonuclease derived from the Gram^+^ bacterium *Bacillus globigii* 26, into the M13KE vector under the control of LacZ promoter, to generate the BGL phage. The BCP1 sequence was subsequently inserted into the *p*Ⅲ gene of BGL to generate the BCP1-BGL phage (Figure 1A). Infection of *E. coli* with these phages, in the presence of isopropyl-D-thiogalactoside (IPTG), leads to the expression of *Bgl* Ⅱ, which causes non-repairable breaks in double-stranded chromosomal DNA and eventual kills the host cells. Western blotting using the anti-his antibody confirmed expression of the *Bgl* Ⅱ protein after infection with BGL and BCP1-BGL, but not SC (a control phage randomly picked from the library) and BCP1, in the presence of IPTG (Figure 1B). At a multiplicity of infection (MOI, refer to the ratio of phage to bacteria) of 10, both BGL and BCP1-BGL effectively inhibited growth of bacteria, consistent with their presumed ability to kill bacteria. SC and BCP1, however, had low but significant activity in delaying bacteria growth, an effect that was attributed to phage infection and amplification, and was observed with any M13 phage capable of infecting bacteria (Figure 1C). Growth of *E. coli* caused a time-dependent increase in endotoxin release to the culture supernatant, but infection with phages, including BGL and BCP1-BGL, did not further increase endotoxin production (Figure 1D). BCP1-BGL exhibited extended blood retention, with a profile that was nearly identical to BCP1, while BGL had the same profile as SC, indicating that the addition of the *Bgl* Ⅱ gene had no effect on blood circulation time of the phage, as expected (Figure 1E). Enhanced accumulation of BCP1 and BCP1-BGL over SC and BGL was also found in the abdominal cavity fluid (ACF) after tail vein injection (Figure 1F), consistent with their long-circulating properties.

### BCP1-BGL exhibited extended anti-bacterial action in a prophylactic model

**Figure 2:**
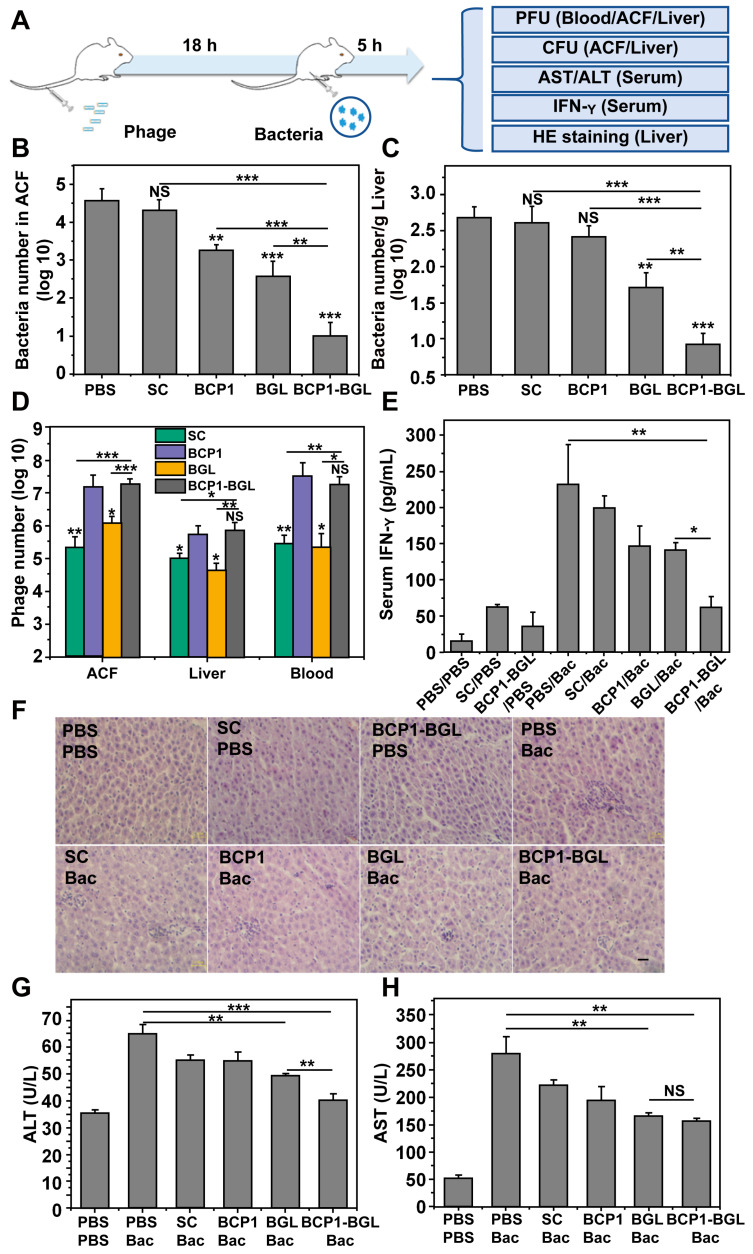
** Prolonged antibacterial activity of BCP1 *in vivo.***(A) Illustration of the prophylactic model to assess the prolonged anti-bacterial action of engineered phages. Rats were administered with 1 × 10^11^ pfu of phage through tail vein injection and, 18 h later, were challenged with injection of 1 × 10^8^ of *E. coli* (OD 600~0.2) into abdominal cavity. After an additional 5 h, various assays were conducted to assess the extent of damage due to bacterial infection. (B) Anti-bacterial efficacy of engineered phages in ACF. Mean ± S.E.M., n = 3, **p < 0.01, ***p < 0.001. (C) Anti-bacterial efficacy of engineered phages in the liver. The number of viable bacteria per gram of liver tissue was determined by colony formation assay. Mean ± S.E.M., n = 3, **p < 0.01, ***p < 0.001. (D) Phage numbers in ACF, liver and blood. Mean ± S.E.M., n = 3, *p < 0.05, **p < 0.01, ***p < 0.001. (E) Evaluation of serum IFN-γ. Bac, bacteria. Mean ± S.E.M., n = 3, *p < 0.05, **p < 0.01. (F) Infiltration of immune cells into the liver, revealed by representative images of liver section following HE staining. Bac, bacteria. Scale bar = 2 μm. (G and H) Liver damage assessed with ALT (G) and AST (H) levels in serum. Bac, bacteria. Mean ± S.E.M., n = 3, **p < 0.01, ***p < 0.001.

Taking advantage of the prolonged phage presence in the ACF, we developed a prophylactic experimental protocol, to assess the extended anti-bacterial efficacy of engineered phages *in vivo* (Figure 2A). Rats were intravenously injected with phages and, 18 h later, challenged with an intraperitoneal injection of *E. coli* and IPTG. The injected bacteria were found to disseminate quickly to other tissues, with the titer of *E. coli* in the ACF being reduced from the initial 10^8^ to less than 10^6^ within 15 min after administration (Figure S1). Assessment of the viable bacterial number in the ACF 5 h post bacterial challenge revealed that BGL, and BCP1 to a lesser extent, were able to reduce bacterial burden (Figure 2B). Importantly, enhanced elimination of bacteria was achieved by BCP1-BGL, and a dramatic and statistically significant improvement over the BGL, as well as the BCP1 phage, was observed. Assessment of bacterial number in the liver revealed the same results, with BCP1-BGL exhibiting the best anti-bacterial efficacy (Figure 2C). No or very few viable bacteria were recovered from the blood at any time after bacteria challenge, including from rats that were not injected with phage (data not shown). We also assessed phage titer in the blood, liver and ACF 5 h after bacteria challenge. In all cases, the number of phages significantly increased after infection as compared to the respective phage titer before infection, and significantly more phages were found in BCP1 and BCP1-BGL-treated rats than in SC and BGL-treated rats (Figure 2D). Significant reduction in serum interferon gamma (IFN-γ) release, elicited by bacterial infection, was also observed in BCP1-BGL-treated animals (Figure 2E). To further evaluate the anti-bacterial efficacy of the engineered phage, we also assessed liver inflammation and damage induced by bacterial infection. Histopathological examination revealed extensive infiltration of inflammatory cells in the liver of rats treated with bacteria, but this inflammatory effect was significantly inhibited by pre-injected BCP1, BGL, and BCP1-BGL, with BCP1-BGL providing the best protection (Figure 2F). In addition, BCP1, BGL and BCP1-BGL also reversed the increase of serum alanine transaminase (ALT) and aspartate aminotransferase (AST) levels resulting from the bacterial infection, with BCP1-BGL phage again exhibiting the best efficacy (Figure 2G-H). Taken together, our results demonstrated that BCP1-BGL phage exhibited prolonged blood circulation and extended anti-bacterial action *in vivo*.

### The BCP1 peptide promoted blood retention of BCP1-BGL phage through interaction with PLT

**Figure 3:**
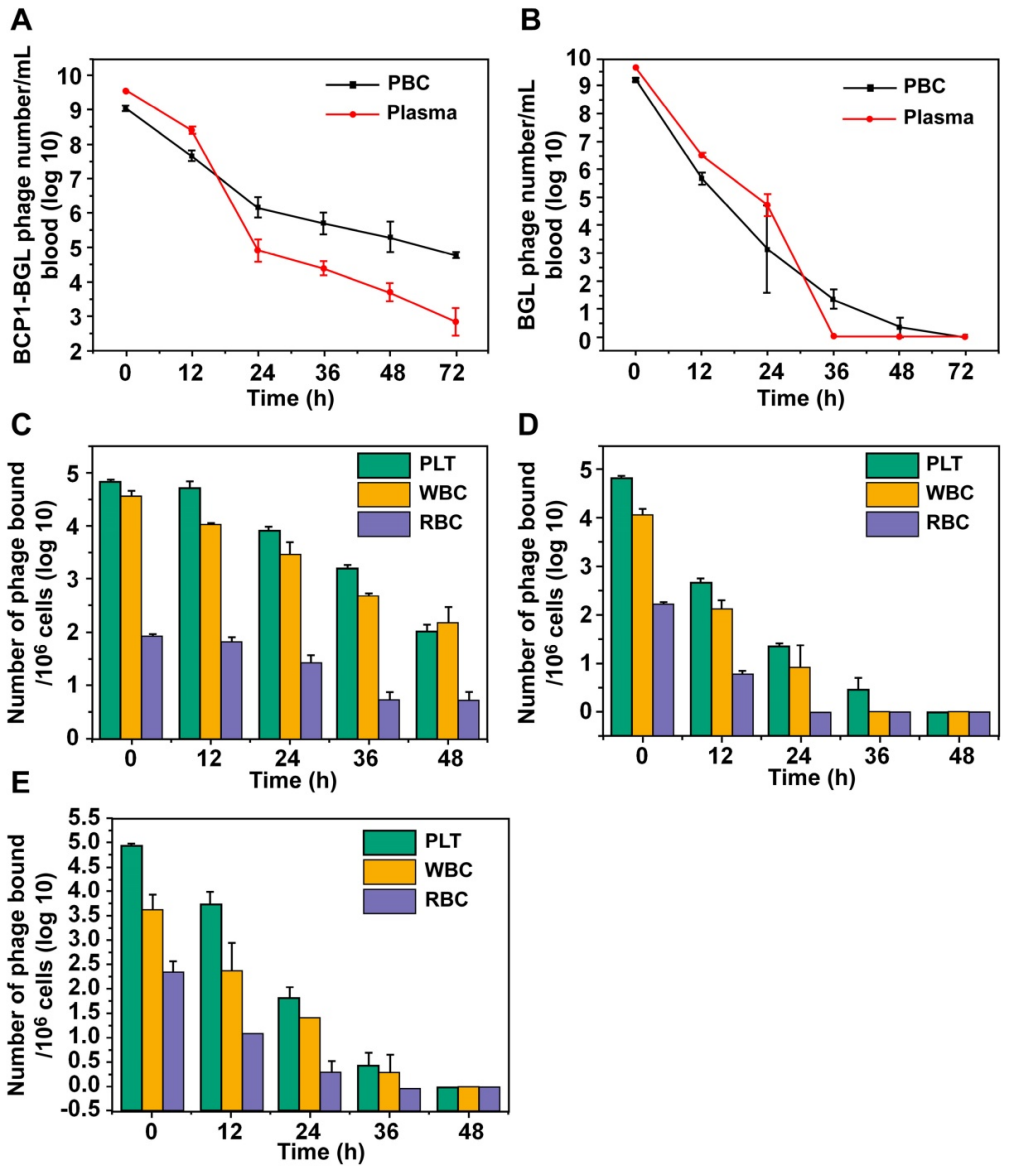
** BCP1 peptide promoted blood retention of phages through interaction with PLT.** (A and B) Relative distribution of phages in the plasma and PBC. BCP1-BGL (A) and BGL (B) (1 × 10^11^ pfu each) were injected into rats through tail vein. Blood taken at different time points was centrifuged to separate plasma from PBC, followed by phage titer determination. Mean ± S.E.M., n = 3, *p < 0.05, **p < 0.01. (C, D and E) Relative binding ability of BCP1-BGL, BGL and TB2-BGL phages to the three PBC components *in vivo*. Blood was withdrawn at various time points after tail vein injection of 1 × 10^11^ pfu of BCP1-BGL, BGL or TB2-BGL, separated into RBC, WBC and PLT by antibody-mediated FACS sorting, and analyzed for the number of bound phages per million cells/PLT. Mean ± S.E.M., n = 3.

In our previous work, we have demonstrated that the circulation-prolonging ability of the BCP1 phage was primarily attributed to its interaction with PBC and particularly PLT in the bloodstream 11. The same circulation-prolonging effect and mechanism would be expected to apply to the BCP1-BGL phage. To verify, we first assessed the relative distribution of phages in the plasma versus the PBC fractions at various times after intravenous administration. More BCP1-BGL phages were found in the plasma than in PBC at 0 h, and this difference decreased by time. After 18 h, we observed an equal number of phages in the plasma and the PBC. By 24 h and beyond, the opposite distribution, with the number of phages in the PBC fraction exceeding that in the plasma, was observed (Figure 3A). Notably, close to 100 thousand phages were still present in 1 mL of blood 72 h post-administration for BCP1-BGL, with over 99% of them bound to PBC. In contrast, a different distribution profile was observed with the BGL phage. The number of BGL phages was higher in the plasma than in the PBC for all the time points except for 36 h, at which almost all of the phages had been cleared (Figure 3B). No phage could be recovered from the blood at 72 h for the BGL phage. To determine which type of blood cells mostly interacted with the phages, we used antibody-mediated FACS sorting to analyze the distribution of phages on the three major components of PBC following *in vivo* administration. When assessed by the number of bound phage per million cells, BCP1-BGL phage exhibited similar high-level binding to PLT and WBC but much lower binding to RBC, with significant binding to PLT and WBC still observed at 48 h post-administration (Figure 3C). Upon taking consideration of the number of particles in the blood (there were approximately 145 times more PLT particles than WBC cells in the blood), we calculated the total number of phages bound to each type of the PBC, revealing that most long-circulating BCP1-BGL phages (93.1%) ended up binding with PLT at 48 h (Table 1), simply because of the overwhelming abundance of PLT in the blood relative to WBC. By contrast, BGL phage diminished rapidly with little and negligible percentage bound on PLT at 48 h (Figure 3D). TB2-BGL, which was identical to BCP1-BGL, except for the fact that the RGD motif within the displayed peptide (CNARGDMHC) was mutated to AGA (CNAAGAMHC), exhibited a binding pattern similar to the BGL phage but different from the BCP1-BGL phage, indicating that the RGD motif was essential for the long-circulating properties of BCP1-BGL (Figure 3E). We, thus, concluded that RGD-mediated interaction between BCP1-BGL and PLT was primarily responsible for the extended blood retention of the BCP1-BGL phage.

### Preparation and characterization of phage-platelet hybrid nanoparticles (PPHNs)

**Figure 4:**
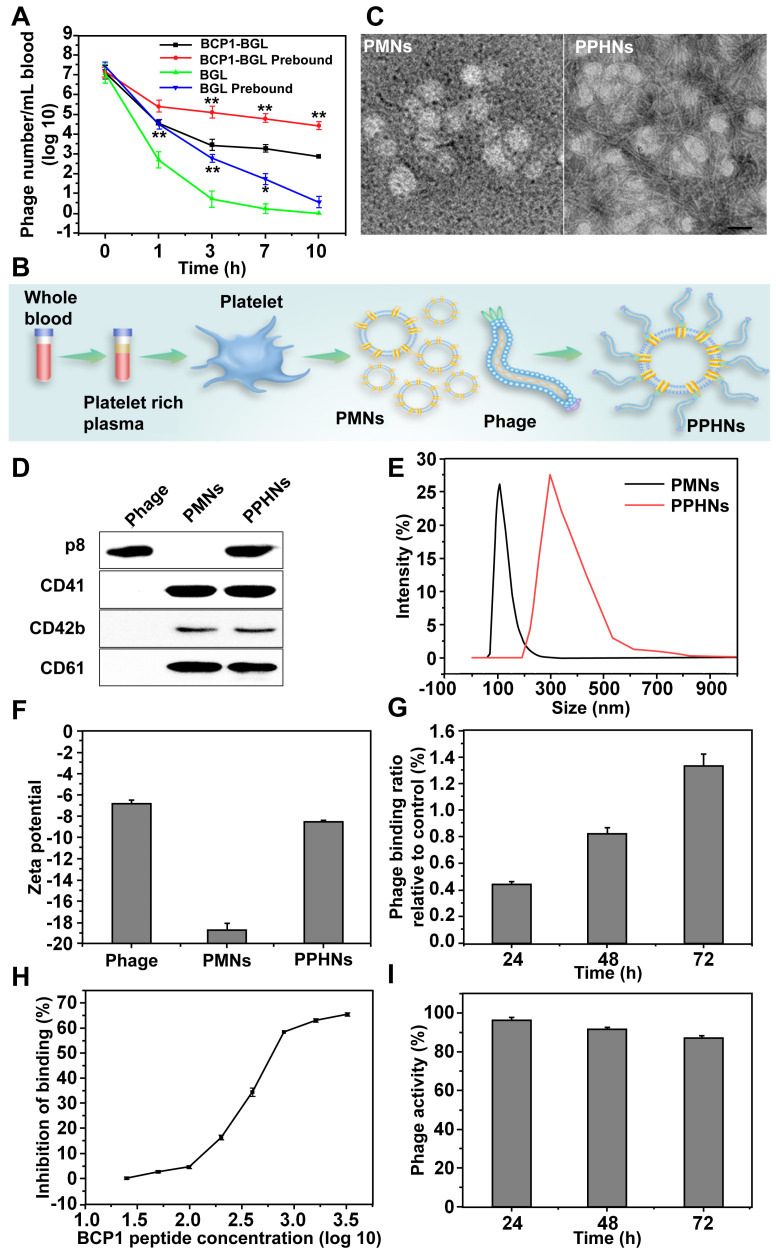
** Preparation and characterization of PPHNs.** (A) Prolonged blood circulation for PBC-bound phage. BCP1-BGL or BGL phages (1 × 10^9^ pfu each), either unbound or pre-bound to PBC, were separately injected into rats and the phage titer in the blood at various time points was determined. Mean ± S.E.M., n = 3, *p < 0.05, **p < 0.01, with BCP1-BGL prebound compared to BCP1-BGL and BGL prebound compared to BGL. (B) Schematic illustration of PPHNs. PLTs extracted from 1 mL of rat blood were used to prepare platelet membrane nanoparticles (PMNs), followed by incubation with 5.2 × 10^12^ pfu of phages. PPHNs were isolated through centrifugation, followed by several washes with PBS. (C) TEM images of PMNs (left) and PPHNs (right) negatively stained with uranyl acetate. Scale bar, 100 nm. (D) Detection of expressed CD41, CD42b, CD61 and P8 in PMNs and PPHNs by western blotting with specific antibodies. (E) Dynamic light scattering analysis of PMNs, PPHNs in water. (F) Zeta potential distribution of phage, PMNs and PPHNs. Mean ± S.E.M., n = 3. (G) Release study of phage bound on PPHNs. Mean ± S.E.M., n = 3. (H) Binding of BCP1-BGL phage to PMNs in the presence of increasing concentration of BCP1 peptide. The number of unbound phages was determined after centrifugation. Mean ± S.E.M., n = 3. (I) Thermal stability of PPHNs. Mean ± S.E.M., n = 3.

While the BCP1-BGL phage exhibited extended blood retention time as compared to the BGL phage, the extent of circulation-prolonging was still non-optimum. Since BCP1-BGL phage achieved prolonged blood circulation primarily through the BCP1-mediated interaction with blood PLT, we assumed that BCP1-BGL pre-bound to PLT would exhibit even better circulation-prolonging effect than the unbound phage. Indeed, this is what we found. BGL phage pre-bound to PBC exhibited prolonged blood circulation, and PBC-bound BCP1-BGL phage had further enhanced blood retention, as compared to their respective phage in the unbound form (Figure 4A). Based on this observation, we designed and fabricated PPHNs, a hybrid nanoparticle composed of PLT membrane fragments and BCP1-BGL, with the phage bound to the membranous nanoparticle through the interaction between the RGD motif of the BCP1 peptide on the phages and the integrin on the surface of PLT membranes (Figure 4B). Approximately 1 × 10^9^ PLTs extracted from 1 mL of rat blood were used to prepare platelet membrane nanoparticles (PMNs), followed by incubation with 5.2 × 10^12^ pfu of phages. After 2 h binding at 25 °C room temperature, PPHNs were isolated through centrifugation, followed by several washes with PBS. The total number of unbound phage in the supernatants and washes was approximately 4.72 × 10^12^, so the number of phages bound to membranous nanoparticles was estimated to be 4.8 × 10^11^. Additionally, the total number of nanoparticles was approximately 3.7 × 10^10^, as determined by the number of plaque-forming units, so we concluded that, on average, each PPHN harbored about 12 phages. Consistent with this, transmission electron microscopy (TEM) imaging revealed spherical nanoparticles with unilamellar membrane structures, wrapped by multiple rod-like phage particles on the surface of PPHNs but not of PMNs (Figure 4C). The presence of PLT membrane proteins (CD41, CD42b, and CD61) and a phage protein (p8) was demonstrated by western blotting, verifying the identity of the PLT membranes and phage (Figure 4D). Dynamic light scattering (DLS) revealed a size of approximately 368 ± 5.22 nm (PDI: 0.54) and 121 ± 3.46 nm (PDI: 0.42) in deionized water for PPHNs and PMNs, respectively (Figure 4E). Three batches of PPHNs were synthesized in this study, exhibiting consistent particle size (356 ± 11.02 nm) (Figure S2). To assess nanoparticle stability, we stored PPHNs particles at 4 °C for 7 days, with the DLS assay performed every day to evaluate aggregation propensity. As shown in Figure S3, PPHNs possessed excellent stability, with minimal size change during the 7-day period. In addition, we also evaluated the stability of PPHNs under lyophilization. Lyophilization is generally regarded as a good practice for long-term storage of nanoparticles, but it may sometimes cause problems such as particle aggregation. To assess this, we lyophilized PPHNs and then measured their size and morphology after reconstitution. Reconstituted PPHNs showed excellent solubility and nearly identical size and morphology as pre-lyophilized PPHNs, indicating no particle aggregation (Figure S4-5). Furthermore, the reconstituted PPHNs retained more than 95% phage infectivity (data not shown). Taken together, these studies indicated that PPHNs could be lyophilized for long-term storage, and could also simply be resuspended in PBS for shorter term use.

To assess the potential effect of the *in vivo* environment on nanoparticle stability, we suspended PPHNs in the rat plasma for various time points and then measured the particle size. Very little change in nanoparticle size was observed (Figure S6). While the unbound phage particles exhibited a slightly negative charge (-6.89 ± 0.64 mV), PPHNs and PMNs showed an increased negative charge of -8.95 ± 0.11 mV and -18.7 ± 1.05 mV, respectively (Figure 4F). The detailed zeta potential distribution data are shown in figure S7. In addition, we also assessed the stability of phage binding to the nanoparticles. The release study showed that the interaction between BCP1-BGL phage and PMNs was stable, with less than 2% phage released after 72 h *in vitro* (Figure 4G). Furthermore, the BCP1 peptide was able to compete away the binding between the BCP1-BGL phage and PMNs in a dose-dependent fashion, with an estimated IC_50_ of 350.8 μM (Figure 4H). In contrast, IC_50_ could not be obtained for the SC peptide and TB2 peptide (Figure S8-9), suggesting that the interaction between the BCP1-BGL phage and platelet membranes was specific and dependent on the BCP1 peptide sequence. PPHNs also had good thermal stability, retaining approximately 90% infectivity after 72 h incubation at 37 °C (Figure 4I).

### Preliminary biosafety evaluation of PPHNs *in vitro* and *in vivo*

**Figure 5:**
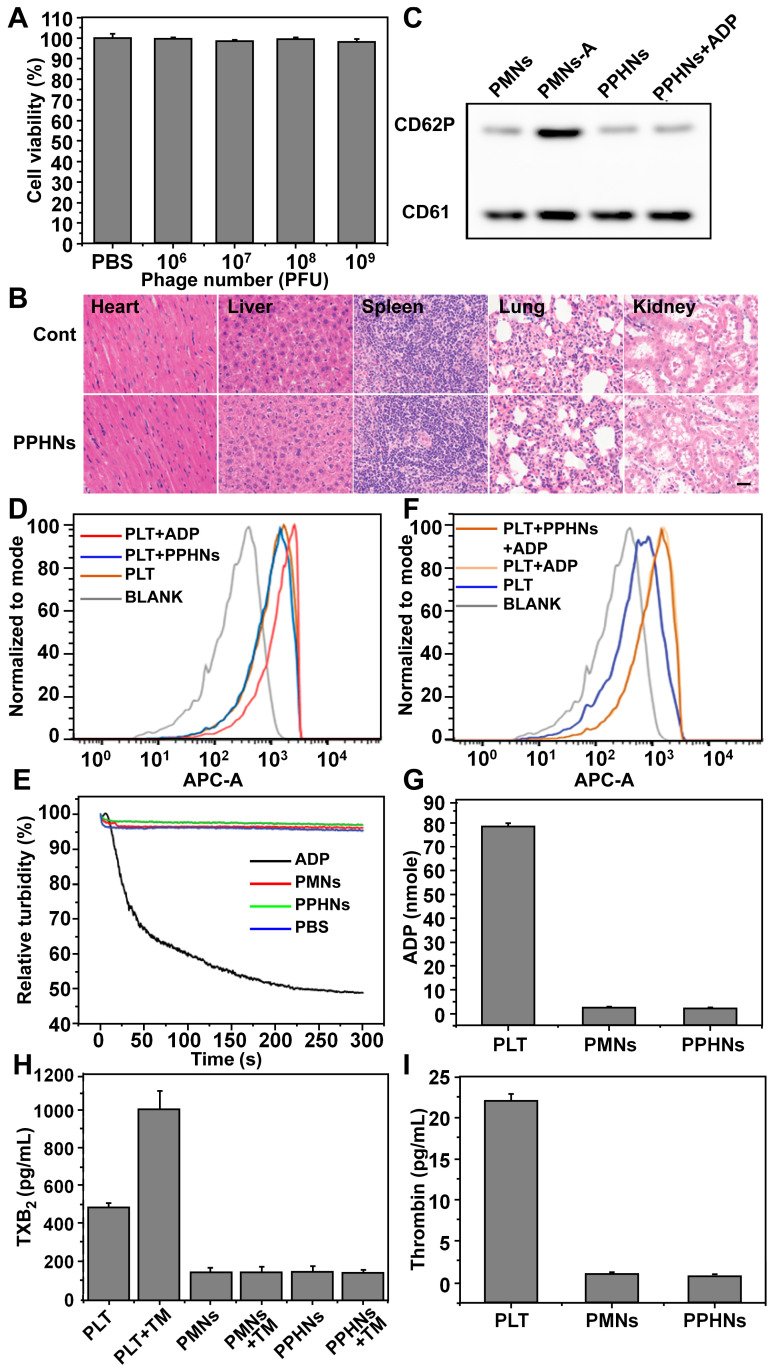
** Preliminary biosafety evaluation of PPHNs.** (A) Cell viability of HUVEC treated with a serial concentration of PPHNs. The horizontal axis stands for the number of phages, which divided by 12 was the number of PPHNs. Mean ± SEM, n = 4. (B) Representative images of organ sections following HE staining after *in vivo* treatment with PPHNs or PBS. Scale bar = 20 μm. (C) Expression of CD62P by western blotting to evaluate platelet membrane activation. PMNs-A (PMNs from active platelets) served as positive control. (D and F) PPHNs did not cause platelet activation (D) and did not affect platelet activation caused by ADP (F). Flow cytometry histograms showing CD62P expression in isolated PLTs. (E) Platelet aggregation assay. Citrate-stabilized PLT rich plasma (PRP) was mixed with PBS, PMNs, PPHNs or ADP followed by spectroscopic examination of solution turbidity. (G-I) Platelet-activating contents ADP (G), thromboxane (TXB2, H) and thrombin (I) in PLT, PMNs, and PPHNs were quantified. TM stands for thrombin. Mean ± SEM, n = 3.

We evaluated preliminary toxicity and bio-safety assessment of PPHNs before examination of anti-bacterial activity. We first performed MTT assay and HO/PI cell death assay in human umbilical cord vein endothelial cell (HUVECs). PPHNs elicited minimal cell viability reduction (Figure 5A) and cell death (Figure S10), demonstrating excellent biocompatibility. HE staining on various rat tissues (heart, liver, spleen, lungs and kidneys) 24 h after tail vein injection of PPHNs (Figure 5B), and no obvious tissue damage was observed following PPHNs treatment.

CD62P, known as P-selectin or granule membrane protein 140, is present in the alpha (a)-granules of resting PLT and is translocated to the plasma membrane after PLT activation. We first evaluated the level of CD62P on the membrane of PMNs and PPHNs by western blotting to assess activation of PLT during PPHNs production. As shown in Figure 5C, minimal CD62P was detected on prepared PMNs and PPHNs, indicating that PLTs were not activated by the extensive manipulation performed for the PLT-phage nanoparticle preparation. In contrast, as a positive control, CD62P expression was detected on PMNs-A (PMNs prepared from activated PLTs). We next addressed whether the as-prepared PPHNs could elicit PLT activation, by employing both a fluorescence-based and a turbidity-based assay. Both assays revealed the same results (Figure 5D-E), with PPHNs eliciting no PLT activation/aggregation. As the positive control, ADP caused robust PLT activation/aggregation, characterized by increased fluorescence and turbidity. Furthermore, we asked whether the as-prepared PPHNs could affect ADP-elicited PLT activation. According to our fluorescence assay, ADP treatment enhanced the fluorescence of PLT, indicative of PLT activation as expected. However, PPHNs did not cause any change on this ADP-elicited fluorescence enhancement (Figure 5F), demonstrating that PPHNs had no effect on ADP-elicited PLT activation.

Finally, we measured the amount of ADP, thromboxane and thrombin, in the membrane of PLT, PMNs and PPHNs. These pro-thrombotic and PLT-activating molecules were present in PLT but were largely removed in the as-prepared PMNs and PPHNs, thus concluding that PPHNs were unlikely to elicit a thrombotic response (Figure 5G-I).

Taken together, the above results provided a good preliminary indication that PPHNs are safe and may be suitable for* in vivo* applications for treating bacterial infection.

### Anti-bacterial activity of PPHNs *in vitro* and *in vivo*

**Figure 6:**
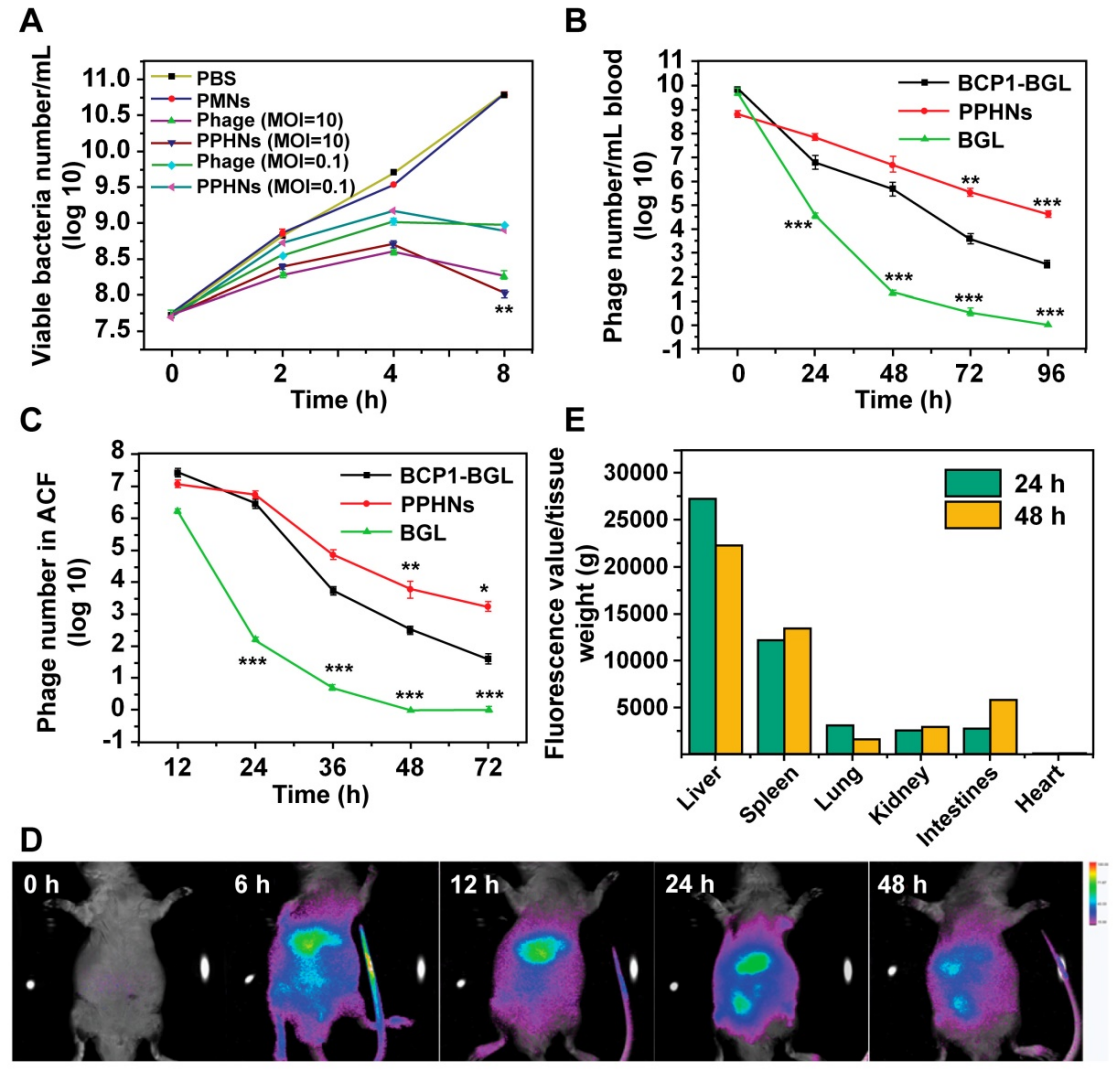
** Long-circulating and bio-distribution of PPHNs.** (A) Antibacterial activity of PPHNs *in vitro* at the MOI of 10 and 0.1. The number of viable *E. coli* was determined at various time points after phage infection with the colony formation assay. Mean ± S.E.M., n = 3. **p < 0.01, with PPHNs compared to BCP1-BGL phage at the MOI of 10. (B) Blood circulation time comparison between the BCP1-BGL phage, PPHNs, and the BGL phage. The same number (1 × 10^11^ pfu) of individual phages was separately injected into rats with phage titers in the blood determined at various time points. Mean ± S.E.M., n = 3. **p < 0.01, ***p < 0.001. (C) Enrichment of long-circulating phage in the ACF. Mean ± S.E.M., n = 3. *p < 0.05, **p < 0.01, ***p < 0.001. (D) *In vivo* fluorescence images of the biodistribution of Cy5.5-labeled PPHNs in rat. (E) *Ex vivo* fluorescence of Cy5.5 of excised organs 24 h and 48 h post-administration.

Each PPHN nanoparticle harbors 12 phages but is presumably able to infect and internalize into just one bacterium. On the other hand, co-entry of multiple infectious phage particles into a bacterium may indicate faster infection kinetics, leading to more rapid cell rupture, release of infectious phages to the medium and re-infection. These opposing factors would govern the anti-bacterial activity of PPHNs and the BCP1-BGL phage. At an equal phage number (but 11 times lower in terms of nanoparticle number for PPHNs), PPHNs would be expected to exhibit lower anti-bacterial activity* in vitro* than the BCP1-BGL phage at low MOI, but comparable and even better performance would be expected for PPHNs over BCP1-BGL at a high (over 1) MOI. Our experimental results were largely in agreement with this hypothesis. At an MOI of 10, a situation where phages are in excess, PPHNs were clearly better than BCP1-BGL in inhibiting bacterial growth, particularly at later time points. As would be expected, PMNs had minimal anti-bacterial activity. In contrast, at a MOI of 0.1, a condition where bacteria are in excess, BCP1-BGL inhibited the growth of *E. coli* more effectively than PPHNs at the early stage, but this difference disappeared slowly over time (Figure 6A), consistent with the notion that PPHNs have faster infection kinetics and would perform better at subsequent rounds of infection. As a verification of phages' infection, we detected the mRNA of *Bgl* Ⅱ in the supernatant of *E. coli* culture infected with BCP1-BGL and PPHNs but not with PBS (Figure S11).

To assess the circulation-prolonging activity of PPHNs we injected the same phage number of BCP1-BGL, PPHNs and BGL into different rats and analyzed phage titers in the bloodstream at various time points. As expected, BCP1-BGL exhibited enhanced blood retention over BGL due to the presence of the BCP1 peptide. The phage tire was for PPHNs than for BCP1-BGL at 0 h, consistent with the fact that the particle number of injected PPHNs was 10 times lower. However, as time went on, the phage titer in PPHNs-infected rats quickly surpassed that in BCP1-BGL-infected rats, with the number of PPHNs being 10 times more at 24 h and 100 more at 96 h over BCP1-BGL (Figure 6B), demonstrating a superior circulation-prolonging activity of PPHNs. The same enhanced accumulation profile of PPHNs was also found in the ACF (Figure 6C). To evaluate bio-distribution, we labeled PPHNs by covalently attaching the Cy5.5 dye molecule to phage particles. Following rat intravenous injection, *in vivo* fluorescence imaging revealed that PPHNs were mainly distributed in the regions around the liver and spleen, but fluorescence intensity gradually diminished over time (Figure 6D). The* ex vivo* fluorescence measurement for the excised organs confirmed these results, as the highest accumulation of Cy5.5 fluorescence was observed in the liver and spleen (Figure 6E).

**Figure 7:**
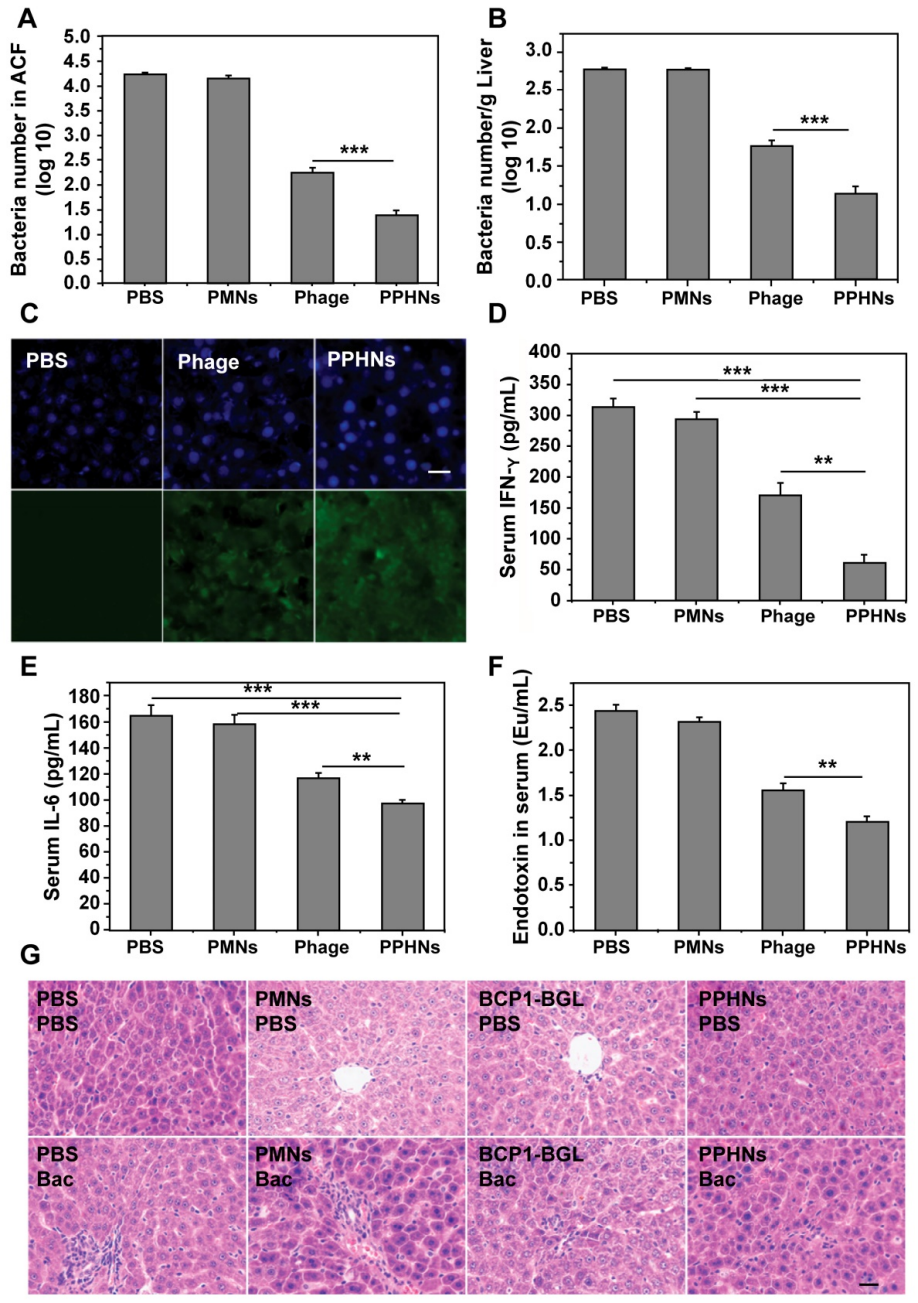
** Anti-bacterial ability of PPHNs in prophylactic infection scheme.** (A) Anti-bacterial efficacy of PPHNs in ACF. Mean ± S.E.M., n = 3. ***p < 0.001. (B) Anti-bacterial efficacy of PPHNs in the liver. The number of viable bacteria per gram of liver tissue was determined with colony formation assay. Mean ± S.E.M., n = 3. ***p < 0.001. (C) Immunofluorescent image of FITC-labeled phages in the liver. Nucleus (blue) was stained with DAPI. Scale bar = 20 μm. (D) Evaluation of serum IFN-γ. Bac, bacteria. Mean ± S.E.M., n = 3. **p < 0.01, ***p < 0.001. (E) Evaluation of serum IL-6. Bac, bacteria. Mean ± S.E.M., n = 3. **p < 0.01, ***p < 0.001. (F) Endotoxin release assay. Endotoxin level in the culture supernatant was determined by ELISA at various time points following phage infection of *E. coli.* Mean ± S.E.M., n = 3. **p < 0.01. (G) Infiltration of immune cells into the liver, revealed by representative images of liver sections after HE staining. Bac, bacteria. Scale bar = 20 μm.

To assess the prolonged anti-bacterial activity of PPHNs, we utilized the same prophylactic infection scheme as described above for BCP1-BGL. Rats were intravenously injected with PPHNs (10^10^ pfu) and, 18 h later, were challenged with intraperitoneal injection of *E. coli* and IPTG. Assessment of the number of viable bacteria in the ACF 5 h post bacterial challenge revealed nearly complete elimination of the bacteria by PPHNs, a dramatic and statistically significant improvement over BCP1-BGL (Figure 7A). Assessment of bacterial number in the liver revealed the same results, with PPHNs showing better anti-bacterial activity than BCP1-BGL (Figure 7B). Accordingly, representative fluorescence microscopy images showed more FITC-labeled phages in the liver of mice treated with PPHNs (Figure 7C). In addition, significant reduction in the level of IFN-γ, IL-6 and endotoxin was also observed in PPHNs-treated mice, as compared to mice treated with BCP1-BGL (Figure 7D-F). Histopathological examination revealed extensive infiltration of inflammatory cells in the liver of rats treated with bacteria, but this inflammatory effect was significantly inhibited by pre-injected BCP1-BGL and PPHNs, with the latter providing better protection (Figure 7G). In addition, PPHNs and BCP1-BGL also reversed the increase of ALT and AST levels resulting from bacterial infection, with PPHNs phage again exhibiting better efficacy than BCP1-BGL (Figure S12-13, respectively).

**Figure 8:**
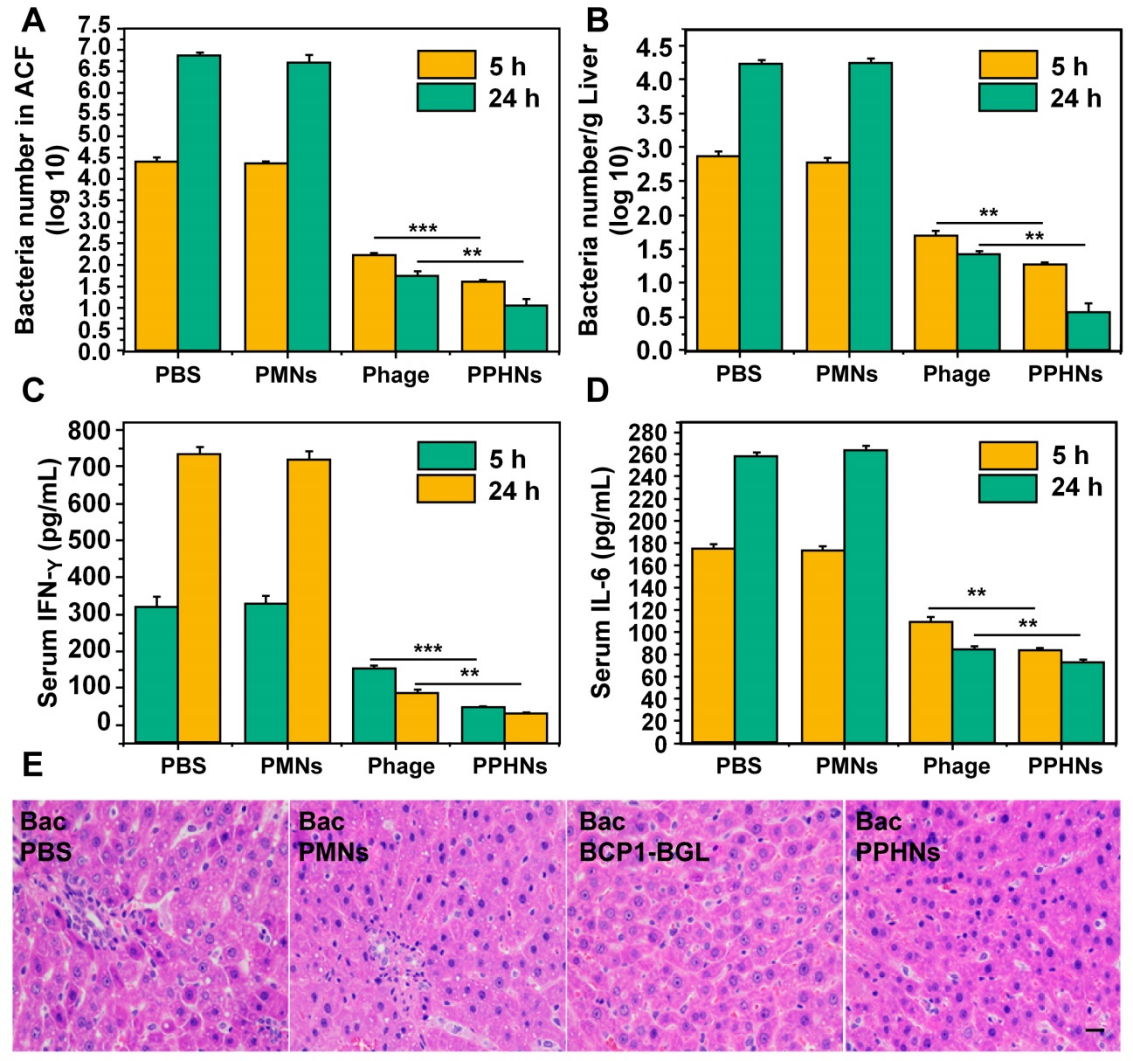
** Anti-bacterial ability in treatment approach.** (A) Anti-bacterial efficacy of PPHNs in the ACF. Mean ± S.E.M., n = 3. **p < 0.01, ***p < 0.001. (B) Anti-bacterial efficacy of PPHNs in the liver. The number of viable bacteria per gram of liver tissue was determined with colony formation assay. Mean ± S.E.M., n = 3. **p < 0.01. (C) Evaluation of serum IFN-γ. Mean ± S.E.M., n = 3. **p < 0.01, ***p < 0.001. (D) Evaluation of serum IL-6. Mean ± S.E.M., n = 3. **p < 0.01. (E) Infiltration of immune cells into the liver, revealed by representative images of liver sections after HE staining. Bac, bacteria. Scale bar = 20 μm.

Furthermore, we have also assessed the anti-bacterial activity of PPHNs after intraperitoneal injection of rats with *E. coli* (10^9^) and IPTG followed by intravenous administration of PPHNs (10^8^ pfu) 15 min later. As shown in Figure 8A-B, both PPHNs and BCP1-BGL, but not PMNs, reduced bacterial burden in the ACF and liver, as assessed 5 h and 24 h post infection. Notably, PPHNs exhibited significantly better anti-bacterial performance than BCP1-BGL at both time points. The same conclusion was reached when the serum levels of IFN-γ and IL-6 were determined (Figure 8C-D, respectively). We also examined histopathologically liver inflammation 24 h after bacterial challenge. While extensive inflammatory cell infiltration in the liver was observed in PMNs-treated rats, BCP1-BGL and PPHNs treatment significantly decreased this infiltration, with a significantly better protection by PPHNs over BCP1-BGL (Figure 8E).

**Figure 9:**
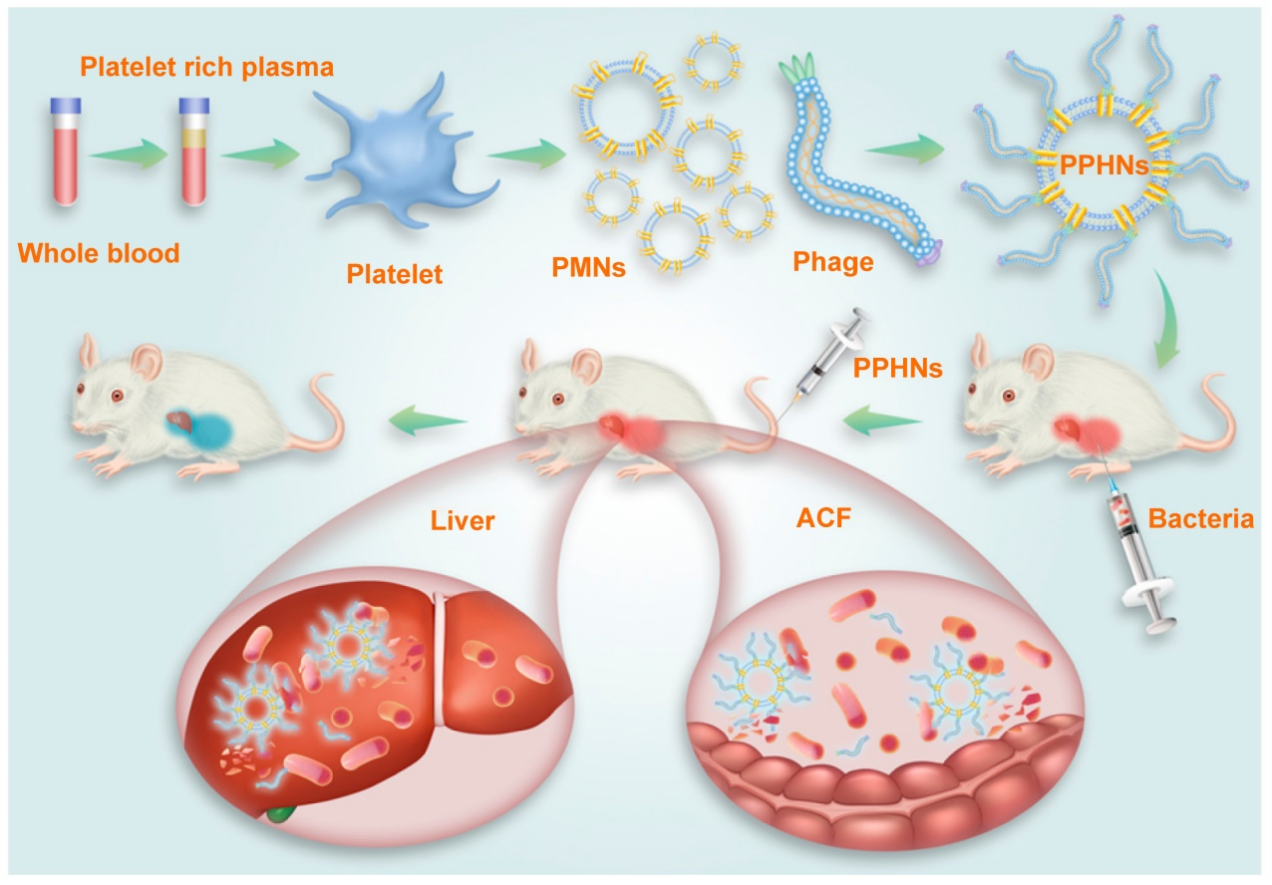
**Schematic illustration of PPHNs' preparation and prolonged anti-bacterial action.** The fabricated biomimetic phage-platelet hybrid nanoparticle (PPHN) was designed through the physical binding of the BCP1-BGL phage to the platelet membrane nanoparticles derived *via* a repeated freeze-thaw. The resulting PPHNs with each spherical membranous nanoparticle harboring approximately 12 rod-shaped phage particles stably bound to its surface, were superior to the BCP1-BGL phages that displayed significantly prolonged anti-bacterial action *in vivo* against *Escherichia coli* infection, exhibited further extended blood retention time and optimal anti-bacterial performance.

Collectively, our results demonstrated that PPHNs exhibited comparable *in vitro* anti-bacterial activity to BCP1-BGL phage but were superior in overcoming bacterial infection *in vivo* in both the prophylactic and treatment approaches. A schematic diagram summarizing the action of PPHNs is shown in Figure 9.

## Discussion

Phage therapy is highly promising for overcoming the paramount problem of antibiotic resistance but faces many hurdles. One major challenge hampering phage therapy is the short blood residence time of bacteriophages, which are rapidly cleared from blood circulation 12. In this work, we have developed a novel strategy for engineering anti-bacterial phages with prolonged blood circulation and improved bactericidal performance. The strategy was achieved in two steps. First, we modified, through genetic engineering, a bactericidal phage with BCP1, a peptide with demonstrated capability in extending blood residence time for nanoparticles 11. The constructed phage, BCP1-BGL, exhibited significantly prolonged circulation time and enhanced anti-bacterial effect in a prophylactic animal model. Compared to other strategies such as natural selection 13, 14 and PEG modification 15, phage engineering by peptide modification provides precise modification, minimal interference on phage activity and convenient mass production. Second, we took advantage of the fact that BCP1-BGL achieves its long-circulating property mostly through binding to platelets, and we constructed PPHNs, a biomimetic BCP1 peptide-anchored PPHN system. PPHNs displayed remarkable long-circulating property in the blood stream, achieving over 100 times higher blood retention than BCP1-BGL 96 h post injection, although the number of administered PPHNs nanoparticles was an order of magnitude lower than the number of administered BCP1-BGL phages. We attributed this incredible circulation-extending ability of PPHNs to two factors. 1) Pre-binding of BCP1-BGL to platelet membrane fragments rendered PPHNs highly effective in preventing phagocytic clearance by the reticuloendothelial system (RES). Hitch-hiking on blood platelets was the mechanism primarily responsible for achieving prolonged circulation for BCP-BGL phages, but phages had to find platelets first in the hostile, dynamic and fast-flowing blood environment. It is conceivable, and even likely, that a sizable percentage of phage particles may have been cleared before they had a chance of landing on platelets for safe transport. Therefore, pre-binding to platelet fragments would ensure that all phage particles can be protected from RES-mediated clearance. 2) Phage inactivation by the complement system is another major factor, in addition to the RES system, that leads to short blood residence time for phages. For a regular phage, one encounter with the complement system may be sufficient to render the phage useless. However, PPHNs contain, on average, 12 phage particles for each nanoparticle. Even when 11 out of the 12 phage particles have been inactivated, the remaining one, in theory, would be still able to infect a bacterial cell. As encountering the complement system is a random event, it would take a long time for all the 12 phage particles to be inactivated. Moreover, co-entry of multiple infectious phage particles into a bacterial cell may indicate faster infection kinetics, leading to more rapid cell rupture, release of infectious phage to the medium and re-infection. Thus, this fail-safe, multi-phage redundant system provided an ideal solution to overcome both the RES-elicited clearance and complement-mediated inactivation, resulting in the outstanding long-circulating characteristics and optimized anti-bacterial activity observed for PPHNs. Our strategy can be easily applied to other therapeutic bacteriophages, as all that needs to be done is to fuse, preferably by genetic engineering, the BCP1 peptide sequence with a protein present on the phage surface. The same procedure for making PPHNs can then be used for preparing biomimetic PPHNs. Given that bacteriophages are generally able to infect only a limited number of bacterial species, this easy-to-adopt strategy may prove to be helpful in combating drug-resistant bacteria.

The RGD motif present within the displayed sequence of the BCP1 peptide was critical for the long-circulating ability of BCP1-BGL, as TB2-BGL, a phage that contained a mutated RGD motif, completely lost the long-circulating property. The target on the platelet membrane is likely to be an integrin, which is a well-known interaction partner for RGD motifs. However, which integrin is responsible for the interaction between BCP1-BGL and platelets is currently unknown. Regardless of the specific interacting integrin, it is likely that it is also present on the membrane of WBCs, but not on RBCs, as high-affinity interaction between BCP1-BGL and WBCs, but not between BCP1-BGL and RBCs, was observed. Further work is required to clarify this issue.

In this study, we have conducted a series of experiments that addressed several issues related to the biosafety of PPHNs. Our results indicated that PPHNs had extremely low toxicity, and neither did they elicit platelet activation *per se*, nor did they affect platelet activation induced by ADP. These results, albeit preliminary, strongly suggested that PPHNs are safe and may be suitable for *in vivo* applications for treating bacterial infection.

## Methods

**Materials:** Ultrapure water (pH 6.7; Milli-Q, Bedford, MA) was used in all experiments. TritonX-100 (T8787), fluorescein isothiocyanate isomer I (FITC, F7250) and ADP Colorimetric/Fluorometric Assay Kit (MAK033) was purchased from Sigma Aldrich (MO, St. Louis, USA). NHS-CY5.5 (A8103) was purchased from APExBIO (Houston, Texas, USA). PEG8000 (A100159), tetracycline (A100422), 5-bromo-4-chloro-3-indolyl-β-D-galactopyranoside (X-Gal, A600083) and IPTG (A100487) were purchased from Sangon Corp (Shanghai, China). Primers were synthesized by Sangon Corp (Shanghai, China). Kits for measuring serum ALT and AST levels were purchased from Rongsheng (Shanghai, China). Antibodies for FACS sorting of blood cells were from BioLegend (San Diego, CA, USA). Rat IFN-γ (CSB-E04579r), IL-6 (CSB-E04640r) ELISA kits and rat Thromboxane B2 (TXB2, CSB-E08047r) ELISA Kit was from CUSABIO (Wuhan, China). Rat anti-CD42b antibody (12860-1-AP), Rat anti-CD61 antibody (18309-1-AP) and mouse anti-his-Tag primary antibody (66005-1-Ig) was from Proteintech Group, Inc (Chicago, IL, USA). ToxinSensorTM Chromogenic LAL Endotoxin Assay Kit was from Genscript (Piscataway, NJ, USA). Rat anti-CD41 antibody (ab181582) was from abcam (Cambridge, U.K.). M13 Bacteriophage (gp8) monoclonal antibody (MAB1948) was from Abnova (Taiwan, China). Rat Thrombin ELISA Kit was from Shanghai Renjie Biotechnology Co. Ltd. (Shanghai, China). All animals used were purchased from Vital River Laboratory Animal Technology Co. Ltd. (Beijing, China).

**Generation of BGL and BCP1-BGL phages:** Cloning of the *Bgl* Ⅱ R-gene in M13 was performed as described previously 28. Briefly, pMRB1 comprising the *Bgl* Ⅱ RM gene was used as a template in a polymerase chain reaction (PCR). The PCR fragment was purified from an agarose gel, eluted with spin column purification (Axygen), digested with *Eco*R Ⅰ and *Hin*d Ⅲ, and ligated overnight at 16 °C with the M13KE vector. Ligation was then introduced into *E. coli* ER2738 competent cells carrying the *Bgl* Ⅱ methylase encoding plasmid PBM1. The engineered phage was propagated in the ER2738 (PBM1) strain expressing the *Bgl* Ⅱ M-gene to ensure “immunity” against the phage encoding the *Bgl* Ⅱ restriction endonuclease and purified following the procedure described above.

**Confirmation of *Bgl***Ⅱ** protein expression:** *E. coli* ER2738 cells (mid-log phase, OD 600~0.5) were infected with phage and induced with IPTG (0.003 mol/L). After 5 h, the supernatant was harvested and concentrated. Proteins were separated by electrophoresis on SDS-polyacrylamide gel to perform western blotting assay following standard procedures.

**Phage amplification:** As we described previously 11, 100 mL LB medium with 20 μg/mL tetracycline and 2 mL *E. coli* ER2738 (OD 600~0.5) was used to amplify phages in a 1000 mL Erlenmeyer flask. After shaking vigorously for 4.5 h at 37 °C, the supernatant was separated by centrifugation (10 min, 10,000 × g), and transferred to a fresh tube. Centrifugation was repeated to remove residual debris, and the supernatant was mixed with 20% PEG/2.5 M NaCl at a ratio of 6:1. After incubation at 4 °C overnight, phages were precipitated by centrifugation at 12,000 × g for 15 min, suspended in 1 mL TBS and re-precipitated with 20% PEG/2.5 M NaCl followed by incubation for 60 min on ice. After repeating the centrifugation, phage pellets were resuspended in 200 µL TBS.

**Determination of plaque-forming units:** As we described previously 11, phages were serially diluted with TBS buffer (50 mM Tris-HCl, pH 7.5, 150 mM NaCl) and mixed with 500 μL *E. coli* ER2738 cells (mid-log phase, OD 600~0.5) in 3 mL top agar (containing 400 μg X-Gal and 500 μg IPTG), followed by pouring onto LB agar plates. After incubation overnight at 37 °C, plaques were counted and multiplied by the dilution factor to obtain phage titers.

**Determination of colony-forming units:** To obtain colony forming units (CFUs), serial dilutions were performed with PBS and spread on LB agar plates. LB agar plates were incubated at 37 °C overnight before counting.

**Comparison of circulation ability:** As we described previously 11, male SD rats (~150 g) were randomly assigned to different groups and were submitted to tail vein injections of 1 × 10^11^ pfu of phages, PMNs or PPHNs. Blood was withdrawn from the heart after treatment at various time points and immediately assayed for phage titer.

**Phage distribution in the plasma and PBC:** Male SD rats (~150 g) were randomly assigned to different groups and were submitted to tail vein injections of 1 × 10^11^ pfu of BCP1-BGL or BGL phages. One milliliter blood was withdrawn from the heart after treatment at various time points and mixed with 100 μL anticoagulant of CPD (16 mM citric acid, 90 mM sodium citrate, 16 mM NaH_2_PO_4_, 142 mM dextrose, pH 7.4). After incubation for 15 min at room temperature, samples were centrifuged at 2,000 × g for 10 min at 25 °C to separate the plasma from PBC. Phage titers in both fractions were assessed immediately, as described previously 11.

**FACS sorting of blood components after *in vivo* phage administration:** Male SD rats (~150 g) were randomly assigned to different groups and were submitted to tail vein injections of 1 × 10^11^ pfu of BCP1-BGL, BGL or TB2-BGL phages. One milliliter blood was withdrawn from the heart after treatment at various time points and was mixed with 100 μL CPD. After incubation for 15 min at 25 °C, samples were centrifuged at 200 × g for 20 min at 25 °C to obtain red blood cells, white blood cells and platelet layers. PLTs were further precipitated by centrifugation at 2000 × g, 4 °C, for 10 min (with 0.2 U/mL apyrase to avoid activation). WBCs were further purified by removing the erythroid cells with erythroid lysis buffer. After incubation with anti-CD16/32 for 10 min on ice in PBS with 1% FBS, cells were sorted after staining with FITC-anti-rat CD45, PE-anti-rat Erythroid Cell and PerCP/Cy5.5-anti-mouse/rat CD42d antibodies. Sorting thresholds were chosen with the aim to exclude the maximum number of unstained maternal cells with minimal loss of stained cells. Cells were sedimented by centrifugation at 2000 × g for 5 min at 4 °C, and immediately assayed for phage titers to detect the number of bound phages per million cells/PLT, as described previously 11.

**Phage release studies:** To examine phage release, 10^6^ PPHNs were diluted in 1 mL PBS (0.1 M, pH 7.4) at 37 °C. The released phage at different time points was determined by the titer of the supernatant, after centrifugation at 40000 × g, 10 min.

**Phage activity studies:** BCP1 phages (1 ×10^7^ pfu) were incubated with 1 mL PBS (0.1 M, pH 7.4) at 37 °C and activity of the phages was detected through their titers at different time points.

**Peptide competitive binding experiment:** 10^6^ pfu of PPHNs were incubated with increasing concentrations of synthetic BCP1, SC or TB2 peptides at 37 °C for 1 h. The number of unbound phages was determined after centrifugation. The inhibition ratio was calculated according to the formula (A-B)/A × 100. (A) The number of bound phages in the absence of peptide. (B) The number of bound phages pretreated with different concentrations of peptide. The value of IC_50_ was calculated with Graphpad.

**Assay for antibacterial activity *in vitro*:** To assess the killing efficiency of the engineered phage, we adopted the method used by S. Hagens 28.* E. coli* MC4100F' grew to OD_600_ = 0.2, and then infected with the respective phages at a different multiplicity of infection (MOI; the ratio of bacterial number to the number of phages), containing 0.003 mol/L IPTG. At various time points after infection, samples were taken from the cultures and immediately assayed for CFUs.

**Endotoxin detection assay:** The endotoxin concentration in *E. coli* strain MC4100F' culture supernatants upon infection was determined with the ToxinSensorTM Chromogenic LAL Endotoxin Assay Kit from Genscript, with absorbance measured at 545 nm. Samples (1 mL) were taken at 0, 1, 2, and 4 h after infection. Relative endotoxin levels were estimated by normalizing against the 0 h value.

**RT-PCR:** *E. coli* ER2738 cells (mid-log phase, OD 600~0.5) were infected with PPHNs or phages and induced by IPTG (0.003 mol/L). After 4 h, the supernatant was harvested and concentrated. mRNA was extracted and RT-PCR assay was performed following standard procedures 29. To determine *Bgl* Ⅱ gene expression, the following primers were used, F: AAATTAGACCGCACTTACATAGGCG; R: TTAATATGTCACGATTGTTCCTCTTTTCC.

**Assay for antibacterial activity *in vivo*:** For the prophylactic infection scheme, as we described previously 11, rats were administered with 1 × 10^11^ pfu of phages (resuspended in 300 μL saline) or PPHNs (10^10^ pfu) through tail vein injection and, 18 h later, were challenged with 1 × 10^8^ cfu of *E. coli* (OD 600 ~0.2) MC4100F' (resuspended in 300 μL saline, mixed with 2 μM IPTG) by intraperitoneal injection, and supplemented with 2 μM IPTG 1 h later. For the treatment approach, rats were intraperitoneally injected with *E. coli* (10^9^ cfu) and IPTG followed by intravenous administration of PPHNs (10^8^ pfu) 15 min later. Rats in both approaches were sacrificed 5 h or 24 h after bacterial challenge, and 1 g liver was aseptically removed and homogenized immediately using glass tissue homogenizers with 1 mL saline solution. To assess the anti-bacterial efficacy of engineered phages in the ACF, 10 mL saline solution was injected into the abdominal cavity of rats. After 3 min, 10 μL ACF was withdrawn and diluted for bacterial number determination. The total number of bacteria in the ACF was estimated by determining the number of viable bacteria for 10 μL ACF and then multiplying by 1000.

**ELISA assay:** Plasma obtained after various treatments was collected and then assayed for IFN-γ and IL-6 according to manufacturer's instructions.

**Histological examination:** Histological examination was performed as reported 30. Briefly, rat livers were taken and inflated with 4% paraformaldehyde, embedded in paraffin and cut at a thickness of 5 μm. For hematoxylin and eosin staining, the slide was first flamed and placed in xylene, and the tissue section was hydrate by passing through decreasing concentrations of alcohol baths (100%, 90%, 80%, and 70%). After staining in hematoxylin for 3-5 min, slides were washed for 5 min with water followed by differentiating in 1% acid alcohol (1% HCl in 70% alcohol) for 5 min and additional washes. Next, slides were stained in 1% Eosin Y for 10 min, washed for 1-5 min, dehydrated in increasing concentration of alcohol, and finally cleared in xylene.

**Determination of serum ALT and AST levels:** Plasma obtained after various treatments was assayed for ALT and AST levels with the automatic biochemical analyzer (Rayto 240, Shenzhen, China) using kits from Rongsheng.

**Platelet membrane derivation:** As reported 31, the blood was centrifuged at 100 g for 20 min at 25 °C to isolate PLTs. The resulting PRP was then centrifuged at 100 × g for 20 min to remove the remaining blood cells. PBS with 1 mM EDTA and 2 mM prostaglandin E1 was added to purify PRP to prevent platelet activation. After centrifugation at 800 × g for 20 min at room temperature, the supernatant was discarded and PLTs were resuspended in PBS containing 1 mM EDTA and protease inhibitors. Platelet suspensions were frozen at -80 °C, thawed at room temperature, and pelleted by centrifugation at 4,000 × g for 30 min. After three freeze-thaw cycles, membranes were washed more than 3 times with PBS mixed with protease inhibitors. The pelleted platelet membranes were suspended in PBS and sonicated in a capped vial for 5 min using a water bath sonicator at a frequency of 42 kHz and a power of 100 W.

**PPHNs preparation and characterization:** 1.5 mL aliquots of platelet solution containing ~1.7 × 10^9^ PLTs were prepared and used to bind with 10^12^ phages. After binding at room temperature for 2 h, PPHNs were purified through centrifugation at 40,000 × g, 4 °C for 30 min. After three washes with PBS mixed with protease inhibitors, PPHNs were suspended in PBS. All supernatants were collected to determine the titer of unbounded phages and the binding rate. The presence of platelet membrane vesicles was verified by size measurement using DLS, western blotting and morphological examination with TEM.

**MTT assay:** HUVEC cells were plated in 96-well plates at a density of 10^4^ cells per well. After overnight incubation at 37 °C, cells were treated with PPHNs at different doses (10^6^, 10^7^, 10^8^ and 10^9^ pfu) for 48 h. Then, the culture media were removed and cells were washed with PBS for three times. MTT (5 mg/mL) was added to each well for another 4 h at 37 °C. The produced formazan was completely dissolved in 150 μL DMSO and absorbance at 490 nm was monitored with ELx800 Absorbance Microplate Reader 32.

Hoechst 33342/PI staining assay: HUVEC cells were plated in 96-well plates at a density of 10^4^ cells per well. After overnight incubation at 37 °C, cells were treated with PPHNs at different doses (10^6^, 10^7^, 10^8^ and 10^9^ pfu) for 48 h followed by staining with Hoechst 33342 and PI (20 μg/mL and 10 μg/mL, respectively) for 10 min and then captured with a fluorescence microscope. PI-positive cells were stained to assess cell death (red) and cell nucleus was strained blue with Hoechst 33342 33.

**Lyophilization of PPHNs:** 50% sucrose buffer was mixed with PPHN solutions to a final sucrose concentration of approximately 10%. PPHN solutions were frozen at -80 °C overnight, followed by lyophilization using the Labconco Free Zone 2.5L Freeze Dry System.

**Phage Label:** FITC-labelled phages were prepared according to the optimized instructions of commercial kits 34. In brief, 200 nM FITC was added to the 50 nM phages' solution in 1 mL carbonate/bicarbonate buffer (100 mM carbonate, pH 8.5). The mixture was incubated at 4 °C overnight and then 1/6 volume of PEG/NaCl buffer was added for 1 h on an ice bath and purified through centrifugation at 10,000 × g, 10 min at 4 °C. The FITC-conjugated phages were washed and concentrated by dialysis. The FITC-phage-PPHNs was prepared using the same method.

For CY5.5 labeled PPHNs preparation, 200 nM NHS-CY5.5 was added to the 50 nM phage solution in 1 mL PBS followed by incubation at 4 °C overnight. Then 1/6 volume of PEG/NaCl buffer was added to the mixture for 1 h on and ice bath and purified through centrifugation 10,000 × g, 10 min at 4 °C. The Cy5.5-conjugated phage was washed and concentrated by dialysis. The Cy5.5-PPHNs was prepared as demonstrated above.

**Platelet-activating molecules examination:** PLT, PMNs and PPHNs with equivalent membrane content were examined for the remaining platelet-activating molecules including thrombin, ADP, and thromboxane, using a rat Thrombin ELISA Kit, ADP Colorimetric/Fluorometric Assay Kit, and Thromboxane B2 (TXB2) ELISA Kit, respectively, based on the manufacturers' instructions.

**Platelet aggregation assay:** Aggregation of PLTs in the presence of PPHNs was assessed using a spectrophotometric method. Aliquots (225 μL) of platelet rich plasma (PRP) and platelets poor plasma (PPP) were first prepared from whole blood with sodium citrate as an anti-coagulant factor and then loaded into a cuvette. After photometric calibration by distilled water and PPP, cuvettes with PRP were placed in the Aggregation Remote Analyzer Module system with the addition of 25 µL PPHNs or PMNs, and immediately monitored for changes in absorbance at 650 nm over time. Platelet aggregation was observed based on reduction of turbidity. As negative and positive controls, PRP was mixed with 25 µL PBS or 25 µL ADP (5.0 μM), respectively.

**Biodistribution study:** In this section, we optimized a previously described method 35. Male SD rats were administered Cy5.5-PPHNs (equivalents of approximately 1 × 10^11^ pfu of phages in 300 μL saline) through tail vein injection. Imaging analysis using the *In Vivo* Xtreme system (Bruker) was performed at specific time points. To examine the fluorescence intensity in various organs, rats were sacrificed at the 24 h and 48 h post injection. Major organs were collected, weighed, and homogenized with an addition of 1:10 (W/V) acidified isopropanol on ice and extracted overnight at -20 °C in the dark. All samples were centrifuged at 14,000 × g for 20 min at 4 °C, and the supernatant fluorescence intensity was measured.

**Statistical analyses:** All data were expressed as mean ± S.E.M. and analyzed with two-tailed Student's t-tests.

**Data availability:** Data supporting the findings of this study are available within the article and the associated Supplementary Information Section. Any other data are available from the corresponding authors upon reasonable request.

## Supplementary Material

Supplementary figures.

## Figures and Tables

**Table 1:** Distribution of phages among PBC components and plasma^a^

TIME	Phage	PLT	WBC	RBC	PLM
0 h	BGL-BCP1	7.8×10^7^ (7.2%)	3.1×10^5^ (<0.1%)	1.2×10^6^ (0.1%)	1.0×10^9^ (92.6%)
	BGL	7.6×10^7^ (3%)	9.9×10^4^ (<0.1%)	1.2×10^6^ (<0.1%)	2.5×10^9^ (96.9%)
	TB2-BCP1	9.9×10^7^ (2.7%)	4.8×10^4^ (<0.1%)	2.0×10^6^ (0.1%)	3.5×10^9^ (97.1%)
12 h	BGL-BCP1	6.3×10^7^ (38.7%)	3.0×10^5^ (0.2%)	6.1×10^5^ (0.4%)	9.8×10^7^ (60.5%)
	BGL	5.3×10^5^ (0.2%)	1.3×10^3^ (<0.1%)	4.4×10^4^ (<0.1%)	3.1×10^8^ (99.7%)
	TB2-BCP1	8.1×10^6^ (8.2%)	8.2×10^3^ (<0.1%)	9.1×10^4^ (0.1%)	9.1×10^7^ (91.7%)
24 h	BGL-BCP1	9.3×10^6^ (81.7%)	8.2×10^4^ (0.7%)	4.9×10^5^ (4.3%)	1.5×10^6^ (13.2%)
	BGL	2.7×10^4^ (33.8%)	1.3×10^2^ (0.2%)	0 (0%)	5.2×10^4^ (66.1%)
	TB2-BCP1	9.1×10^4^ (44.3%)	3.4×10^2^ (0.2%)	1.6×10^4^ (7.9%)	9.8×10^4^ (47.6%)
36 h	BGL-BCP1	4.6×10^6^ (93.1%)	2.9×10^4^ (0.6%)	2.1×10^5^ (4.3%)	9.9×10^4^ (2.0%)
	BGL	3.8×10^3^ (100.0%)	0 (0%)	0 (0%)	0 (0%)
	TB2-BCP1	4.2×10^3^ (99.3%)	3.1×10^2^ (0.7%)	0 (0%)	0 (0%)
48 h	BGL-BCP1	6.5×10^5^ (93.1%)	4.2×10^3^ (0.6%)	4.2×10^4^ (6.1%)	2.0×10^3^ (0.3%)
	BGL	0 (0%)	0 (0%)	0 (0%)	0 (0%)
	TB2-BCP1	0 (0%)	0 (0%)	0 (0%)	0 (0%)

^a^Shown is the number of phage associated with the various blood components in 1 mL of blood, with the relative percentage shown in parentheses. Calculation was based on the number of phage per million blood cells/PLT (Figure 3C-E), The number of blood cells/PLT per mL of blood, taken from ref 27. (PLT, 1.14 ×10^9^; WBC, 7.84 × 10^6^; RBC, 7.01 ×10^9^). PLM, plasma.

## Abbreviations

ACF: abdominal cavity fluid
ALT: alanine transaminase
CTCs: circulating tumor cells
DLS: dynamic light scattering
HUVEC: human umbilical cord vein endothelial cell
IPTG: isopropyl-D-thiogalactoside
MOI: multiplicity of infection
PBC: peripheral blood cells
PCR: polymerase chain reaction
PMN: platelet membrane nanoparticles
PPHN: phage-platelet hybrid nanoparticle
PPP: platelets poor plasma
PRP: platelet rich plasma
RBC: red blood cell
RES: reticuloendothelial system
TEM: transmission electron microscopy
WBC: white blood cells
