# Supplementary material for: A blood circulation-prolonging peptide anchored biomimetic phage-platelet hybrid nanoparticle system for prolonged blood circulation and optimized anti-bacterial performance
Source: Theranostics. 2021 Jan 1;11(5):2278–96. doi: 10.7150/thno.49781 (PMC7797693; doi:10.7150/thno.49781)
Supplement: Supplementary file 1 — Supplementary figures. [file thnov11p2278s1.pdf]

Supplementary Information

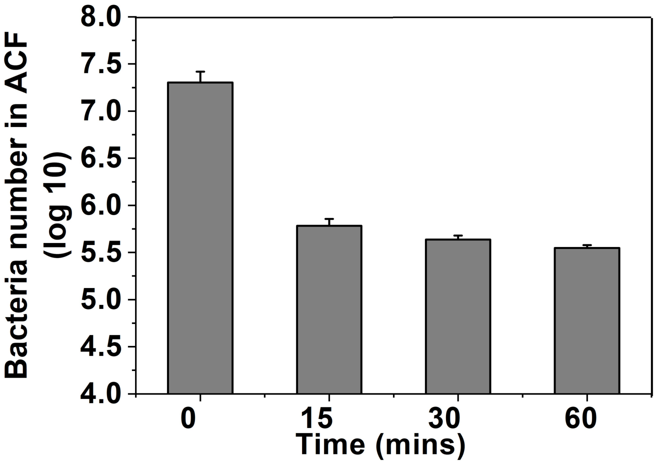

Figure S1. Assessment of viable bacteria numbers in the ACF within 1h after challenge. 10 mL of saline were injected into abdominal cavity of rats at the various times after bacteria challenge, and total number of bacteria in ACF was estimated by determining the number of viable bacteria for 10 μL of ACF and then multiplying by 1000. Mean ± S.E.M., n = 3.

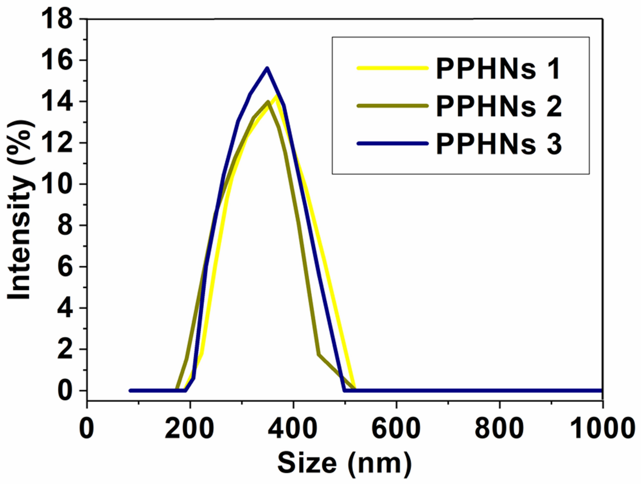

Figure S2. Dynamic light scattering analysis of three batches of PPHNs.

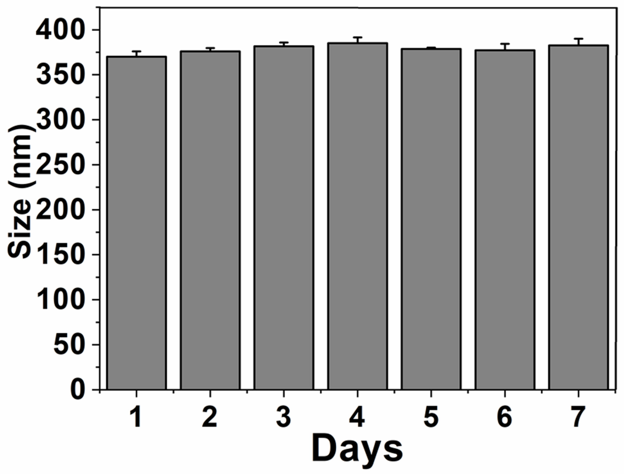

Figure S3. Long-term stability study of PPHNs. The PPHNs were stored at 4 °C for 7 days, and DLS assay was performed every day to evaluate the aggregation propensity.

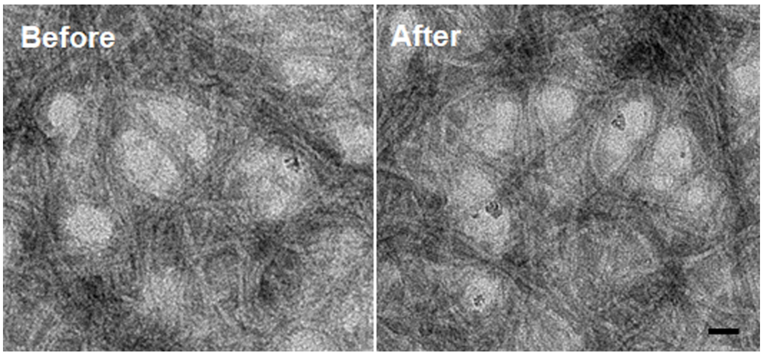

Figure S4. TEM images of PPHNs before (left) and after (right) lyophilization. PPHNs negatively stained with uranyl acetate. Scale bar, 50 nm**.**

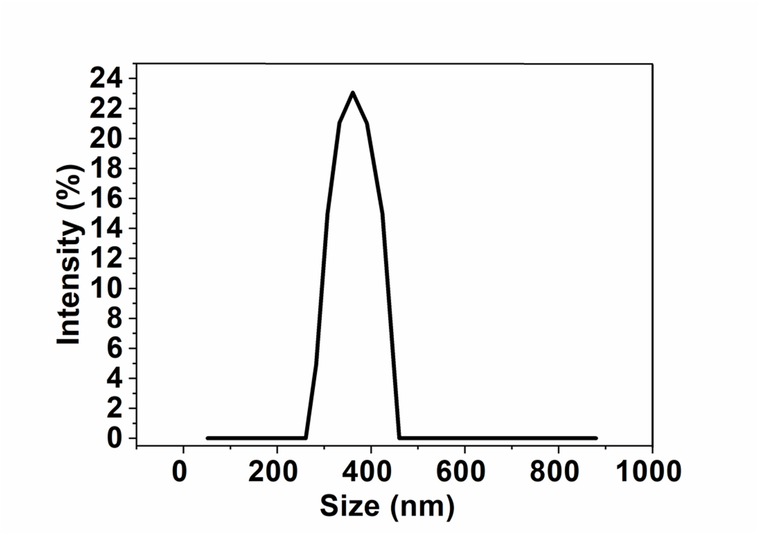

Figure S5. Dynamic light scattering analysis of PPHNs after lyophilization.

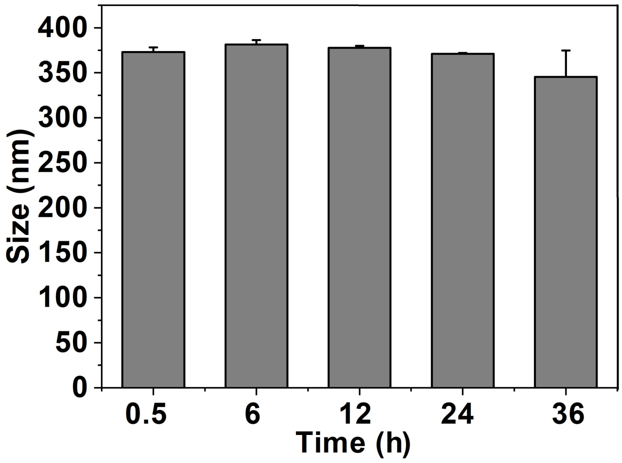

Figure S6. Dynamic light scattering analysis of PPHNs in plasma. PPHNs were dissolved in rat plasma to mimic the *in vivo* environment for different times and conducted dynamic light scattering analysis.

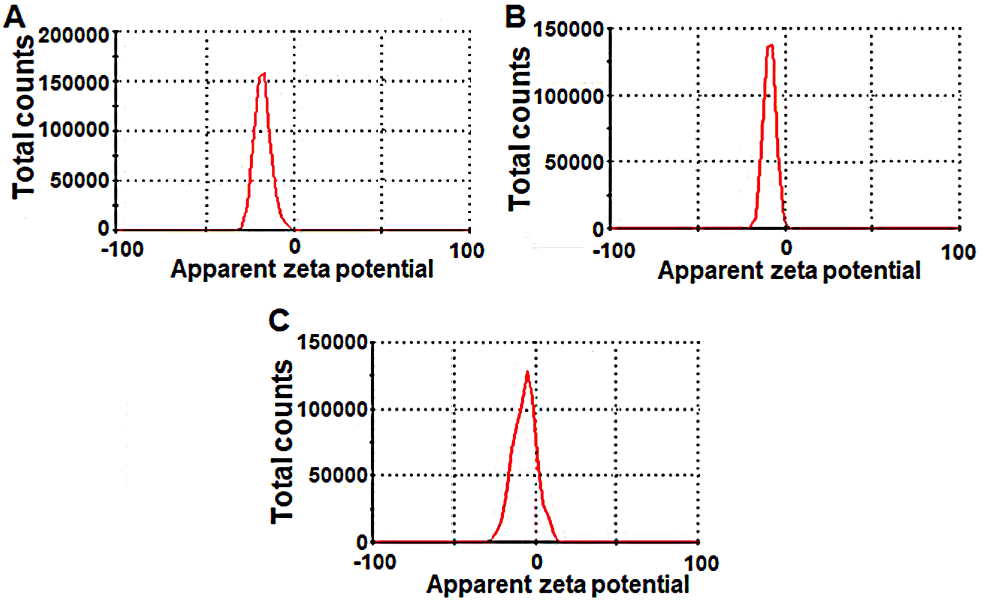

Figure S7. Zeta potential distribution data of PMNs (A), PPHNs (B) and phage (C).

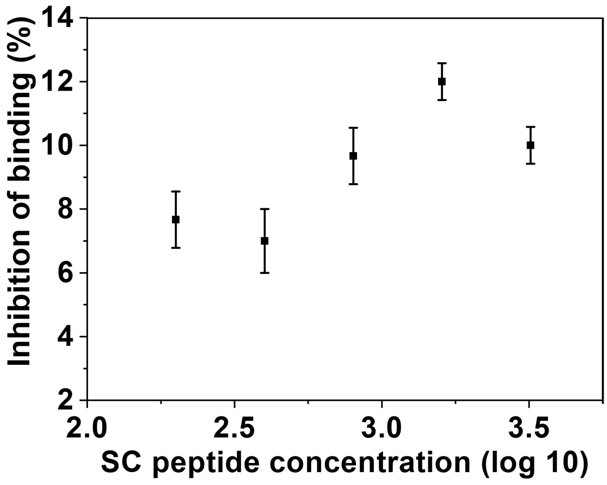

Figure S8. Binding specificity assay. BCP1-BGL phage was incubated with PMNs in the presence of increasing concentration of SC peptide. The number of unbound phage was determined after centrifugation. Mean ± S.E.M., n = 3.

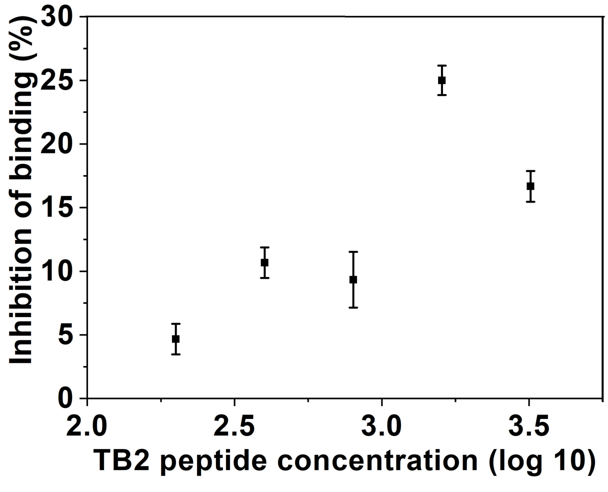

Figure S9. Binding specificity assay. BCP1-BGL phage was incubated with PMNs in the presence of increasing concentration of TB2 peptide. The number of unbound phage was determined after centrifugation. Mean ± S.E.M., n = 3.

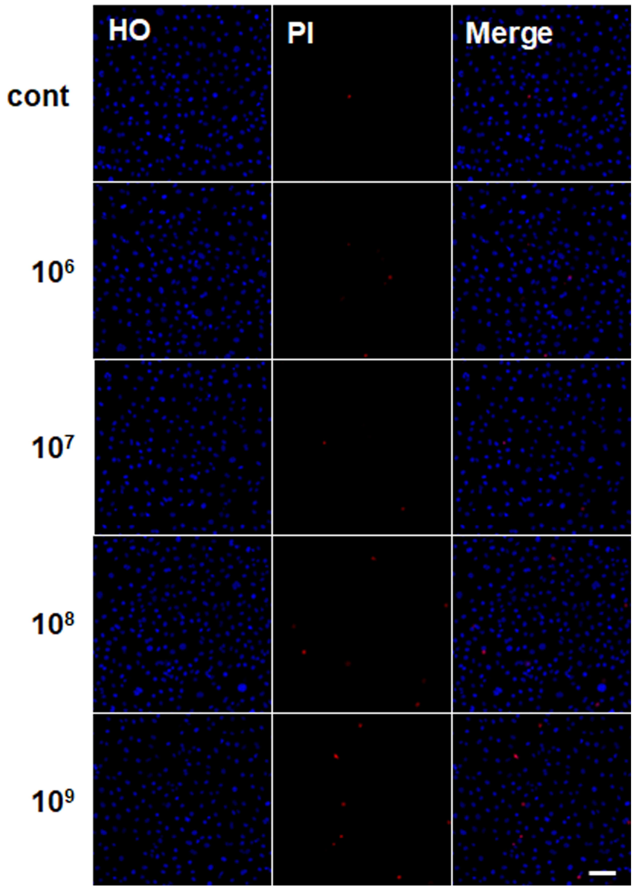

Figure S10. Viability detection of HUVCE cells treated with a serial concentration of PPHNs using HO/PI assay. Mean ± SEM, n = 4. Nucleuses (blue) were stained by Hoechst. Dead cells were stained red by propidium iodide. Scale bar = 20 μm.

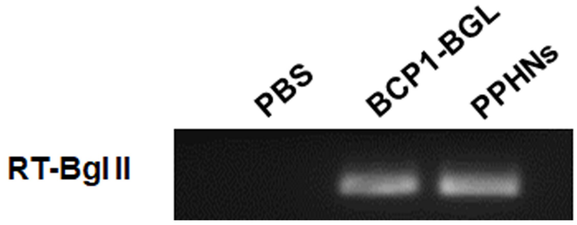

Figure S11. ***Bgl*** **II protein expression induced by IPTG.** *E. coli* ER2738 was infected with PPHNs or BCP1-BGL phage and induced by IPTG (0.003 mol/L). After 4 h, the supernatant was harvested and concentrated. The mRNA was extracted and performed RT-PCR assay followed by agarose gel electrophoresis.

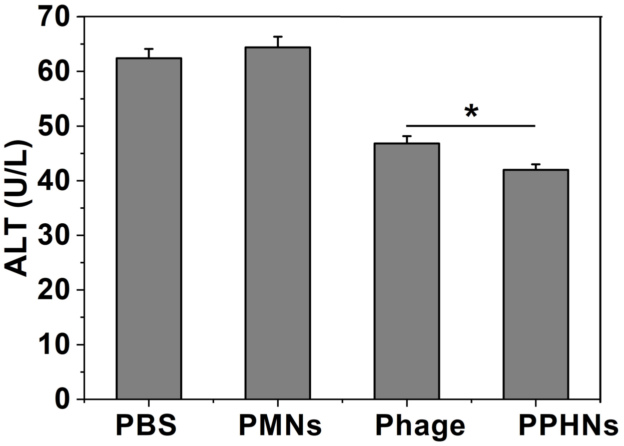

Figure S12. Assessment of serum alanine transaminase (ALT) levels. ALT levels detection after bacterial infection. Mean ± S.E.M., n = 3. $*p < 0.05$.

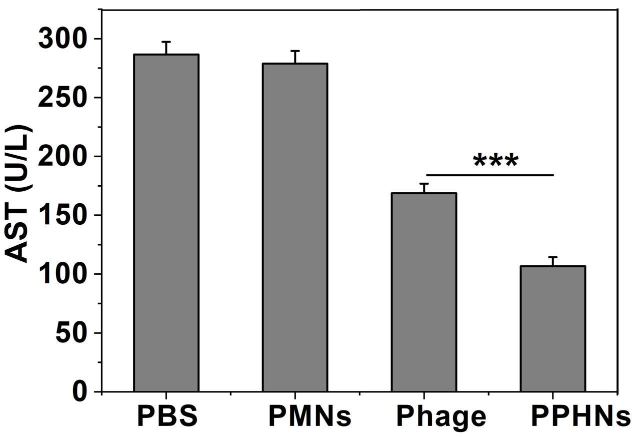

Figure S13. Assessment of serum aspartate aminotransferase (AST) levels. AST levels detection after bacterial infection. Mean ± S.E.M., n = 3. ***$p < 0.001$.
